# Standardization of microbiome studies for urolithiasis: an international consensus agreement

**DOI:** 10.1038/s41585-021-00450-8

**Published:** 2021-03-29

**Authors:** Naveen Kachroo, Dirk Lange, Kristina L. Penniston, Joshua Stern, Gregory Tasian, Petar Bajic, Alan J. Wolfe, Mangesh Suryavanshi, Andrea Ticinesi, Tiziana Meschi, Manoj Monga, Aaron W. Miller

**Affiliations:** 1 Glickman Urological and Kidney Institute, Cleveland Clinic, Cleveland, OH USA; 2 The Stone Centre at VGH, Department of Urologic Sciences, University of British Colombia, Vancouver, BC Canada; 3 Department of Urology, University of Wisconsin-Madison School of Medicine and Public Health, Madison, WI USA; 4 Department of Urology, Albert Einstein College of Medicine, Bronx, NY USA; 5 Division of Urology, The Children’s Hospital of Philadelphia, Philadelphia, PA USA; 6 Department of Microbiology & Immunology, Loyola University Chicago, Maywood, IL USA; 7 Yenepoya Research Centre, Yenepoya University, Mangalore, India; 8 Geriatric-Rehabilitation Department, Azienda Ospedaliero-Universitaria di Parma, Parma, Italy; 9 Department of Medicine and Surgery, Universitaria di Parma, Parma, Italy; 10 Department of Urology, University of California San Diego School of Medicine, La Jolla, CA USA; 11 Department of Cardiovascular and Metabolic Sciences, Cleveland Clinic, Cleveland, OH USA

**Keywords:** Renal calculi, Microbiome

## Abstract

Numerous metagenome-wide association studies (MWAS) for urolithiasis have been published, leading to the discovery of potential interactions between the microbiome and urolithiasis. However, questions remain about the reproducibility, applicability and physiological relevance of these data owing to discrepancies in experimental technique and a lack of standardization in the field. One barrier to interpreting MWAS is that experimental biases can be introduced at every step of the experimental pipeline, including sample collection, preservation, storage, processing, sequencing, data analysis and validation. Thus, the introduction of standardized protocols that maintain the flexibility to achieve study-specific objectives is urgently required. To address this need, the first international consortium for microbiome in urinary stone disease — MICROCOSM — was created and consensus panel members were asked to participate in a consensus meeting to develop standardized protocols for microbiome studies if they had published an MWAS on urolithiasis. Study-specific protocols were revised until a consensus was reached. This consensus group generated standardized protocols, which are publicly available via a secure online server, for each step in the typical clinical microbiome–urolithiasis study pipeline. This standardization creates the benchmark for future studies to facilitate consistent interpretation of results and, collectively, to lead to effective interventions to prevent the onset of urolithiasis, and will also be useful for investigators interested in microbiome research in other urological diseases.

## Abstract (web summary)

Metagenome-wide association studies have led to the discovery of potential interactions between the microbiome and urolithiasis, but a lack of standardization has raised questions about the reproducibility, applicability and physiological relevance of metagenome-wide association study data. This Consensus Statement describes the formation of the first international consortium for microbiome in urinary stone disease — MICROCOSM — and the outcomes of a consensus meeting to develop standardized protocols for microbiome studies and generate a data repository, which are both publicly available via a secure online server.

## Introduction

The role of the microbiome in health and disease is gaining increasing attention and research focus. Advances in high-throughput enhanced culture techniques (metaculturomics) and culture-independent methods (such as 16S ribosomal RNA (rRNA) gene sequencing and shotgun metagenomic sequencing) will only expand the depths to which we can explore these associations and potential causal links. Large-scale projects, such as the American Gut Project^1^, benefit from sampling the microbiome from thousands of individuals, complete with extensive metadata that include the status of numerous diseases. However, such large-scale projects are hindered by a lack of nuance in the information ascertained about specific diseases — for example, of 524 metadata terms associated with the American Gut Project, one term refers to kidney disease, three terms relate to diabetes, and none relates to urolithiasis or other urological diseases, despite numerous reports of potential links between the gut microbiome and urological health^2–5^.

Urolithiasis, the prevalence of which has increased from 3.2% in 1976 to 10.1% in 2016 (ref.^6^), has undergone a rapid shift in epidemiology over the past 25 years. The age at onset is becoming lower and the gender gap narrowing^7^. This disease creates a $10 billion burden on the health-care system in the USA^8^. Urolithiasis is considered a multifactorial disease with numerous disease phenotypes manifesting in different stone types that include calcium oxalate monohydrate, calcium oxalate dihydrate, calcium phosphate, struvite, uric acid, cystine and drug-induced stones^9^. Several environmental and metabolic risk factors can contribute to the onset of urolithiasis such as diet, metabolic disorders, urine composition and volume, urinary tract infections and genetic predisposition^9^. Variability in host genome and the host’s gut or urinary microbiome can affect whether stones develop and stone composition^10–13^. Given the complex nature of urinary stones, determining whether and how the microbiome contributes to the onset of stones requires a nuanced approach.

Numerous culture-independent microbiome studies have been published since 2016 (refs.^2,3,13–19^) in an attempt to address the question of whether the microbiome contributes to the onset of urolithiasis. Although published studies comparing the microbiome from patients with stones with that of healthy controls share some similar results, clear differences are apparent^20^. For instance, whereas some microbial taxa, such as *Prevotella* or *Bacteroides*, are consistently found to be associated with urolithiasis, studies are inconsistent on whether the composition of the gut microbiota as a whole is associated with the disease. Thus, questions remain about the reproducibility, applicability and physiological relevance of these metagenome-wide association studies (MWAS). In particular, whether differences in the results between studies are due to experimental factors (such as sample collection, storage, DNA extraction, sequencing or data analysis) or biological biases (such as geography, ethnicity, stone phenotype or some other regional factors) is unclear.

To answer the fundamental questions associated with the role of the microbiome on stone disease, experimental biases must be minimized between studies to enable direct comparison, even when the objectives of the study vary^21^. Thus, protocols for urolithiasis-associated MWAS must be standardized as much as possible, as minor differences in protocol can drastically affect the downstream analysis of high-throughput sequencing data^21^. As such, a consensus for each step of the experimental pipeline is needed among investigators in the field to enable uniform and meaningful comparisons between studies, while maintaining the flexibility for study-specific objectives.

The objectives of this project are to develop the first international consortium focused on microbiome–urolithiasis research (MICROCOSM — MICRObiome contributions on the Complexity Of the Stone Matrix) and to come to an expert consensus amongst investigators in the field regarding standardized protocols that are recommended for all future clinical MWAS associated with urolithiasis. Formation of MICROCOSM will facilitate the implementation of this expert consensus agreement on protocol standardization to minimize the experimental biases and barriers associated with microbiome research, while enabling flexibility of study-specific objectives. This Consensus Statement also produced a central repository for relevant protocols, raw metagenomic data and real-time meta-analyses of MWAS associated with stone disease, which is available online for free (Box 1). This resource will enable investigators to compare datasets across studies, even if the objectives of the study are different, and will be particularly useful for investigators who do not currently have the necessary equipment or expertise to carry out this type of research at their own institution. With any area of research, having appropriate protocols for the research is one of the biggest barriers to performing the studies. This centralized repository will provide a robust foundation for future multi-institutional studies and will facilitate the comparisons of results across multiple independent studies to answer crucial clinically relevant questions. As such, this Consensus Statement is intended to make collaborative research possible. By standardizing protocols and making them widely available, we seek to ensure that investigators are performing the crucial steps of an MWAS in the same manner, enabling meaningful merging of the data.

Box 1 Links to the standardized protocols and updated meta-analysis results for urolithiasis MWAS Server where all protocols and current metagenome-wide association studies (MWAS) data are housed. Free account required for login:

https://www.lerner.ccf.org/cms/miller/uscd/app/

Link to protocols to conduct MWAS for urolithiasis:

https://www.lerner.ccf.org/cms/miller/uscd/app/?route=documents/type&protocols

Link to mapping file templates. These are required to collect consistently defined clinical metadata for MWAS:

https://www.lerner.ccf.org/cms/miller/uscd/app/?route=documents/type&templates

Link to the most to up-to-date meta-analysis of currently available MWAS data for urolithiasis:

https://www.lerner.ccf.org/cms/miller/uscd/app/?route=documents/type&results

## Methods

A panel of experts with experience in stone disease, microbiology, nutrition and microbiome analysis was convened to form the MICROCOSM consortium. The 12 members of the consortium identified the areas requiring standardization and set out their recommendations.

### Consensus development process

The consensus process was developed using the following steps: identification and recruitment of an expert panel for creation of the MICROCOSM consortium, identification of key issues and protocols requiring standardization, development of protocols and statements based upon best available evidence, and consortium consensus based on a modified Delphi technique at a consortium group meeting^22^.

Members were identified for the consortium based on documented expertise and publications in the field of microbiome and urolithiasis research. Specifically, a comprehensive literature search of PubMed using the keywords “microbiome” AND “urolithiasis” OR “urinary stone disease” OR “nephrolithiasis” was performed to identify relevant clinical microbiome studies associated with urinary stones published before July 2020. The corresponding authors of the publications were then contacted and invited to participate in the consortium. Of 12 individuals contacted, 10 agreed to participate in the consortium. One individual declined the invitation and one individual failed to respond.

Initial correspondence was aimed at identifying the key issues and areas that required consensus agreement. After receiving feedback on this from all members, the next round of correspondence involved the circulation of all protocols for sample collection, storage and processing (for stool, urine and stone samples), DNA extraction methods, and sequencing platform and analysis. Feedback on protocols was collected, addressed and re-circulated to the consortium. Multiple rounds of feedback were performed for all protocols until a consensus agreement was received when >80% of respondents expressed either a strong agreement or agreement with some minor reservation.

An inaugural meeting of most MICROCOSM members was held in person on 7 December 2019 at the StoneLab Scientific Symposium in Linthicum, MD, USA for refinement and further discussion of the consensus points. This meeting site was selected as most consortium members were in attendance at the existing Symposium, but no other affiliation exists between the consortium and the StoneLab Symposium. Meeting and discussion with consortium members who were not present in person occurred via teleconferencing and email correspondence.

### MICROCOSM consensus panel

The expert panel that formed the MICROCOSM consortium comprised 12 experts, with members from academic institutions in North America, Europe and Asia. Members included five urologists, two internal medicine physicians specializing in kidney stone disease, four microbiologists and one dietician, all of whom have documented research experience with both urolithiasis and the microbiome. All included consortium members are co-authors of this Consensus Statement.

The expert panel identified six categories that required standardization: metadata collection; sample collection; preservation, storage and processing of samples; DNA extraction; high-throughput sequencing including the sequencing methodology, platform and data analysis; and metaculturomics.

Notably, this consensus is based on the best available evidence at the time of writing and methods can change with developing science; thus, protocols might need to be updated in the future. As such, consortium members will meet to discuss updating protocols as needed.

## Results

Through the consensus procedures described above, the MICROCOSM expert panel formulated recommendations for all experimental steps in MWAS, from metadata and sample collection to sample analysis and metaculturomics.

### Metadata collection

Collection of appropriate clinical metadata is crucial to enable correlation of research laboratory findings with different clinical parameters and provide the level of nuance required for a complex disease such as urolithiasis. Although the consortium members recognize that the metadata collected is, in part, based on the specific study objectives, consortium members identified urolithiasis-specific metadata that should be collected, which was grouped into sample variables, patient variables, stone variables, antibiotic variables, past medical history, gastrointestinal variables, laboratory values and dietary history (Table 1). To facilitate metadata collection, MICROCOSM has developed a structured questionnaire incorporating most of the variables that would not be readily obtained from the patient’s medical record and created template files for recording these metadata, which are available for download (Table 1, Supplementary information 1). This comprehensive questionnaire is a useful resource for investigators wishing to start data collection for their patients and is useful for clinical as well as research purposes. The metadata categories and questionnaire provided are intended to serve as a baseline and can be modified for specific study objectives.

**Table 1:** List of MICROCOSM Consensus Agreement protocols available

Protocol	Description	Location of full information in Supplementary information or server
Instructions for MWAS	Details the objectives of the consensus, the protocols available and how to use the server/automated analytical pipeline	Supplementary data pages 1–6
***Metadata collection***		
Metadata definitions for 16S and shotgun studies	Details the specific terms and definitions to use for clinical data to be consistent across studies	Supplementary data pages 7–10 Table S1
MICROCOSM questionnaire for patients	A sample questionnaire that can be modified and included as part of an IRB application at the investigator’s home institution	Supplementary data pages 30–50
***Sample collection***		
Mid-stream voided urine sample collection protocol for patients	A protocol that details how patients can self-collect urine samples and ship them to a receiving institution	Supplementary data pages 11–14
Urine collection at time of procedure for physicians	A protocol that details the collection of upper urinary tract urine by physicians	Supplementary data pages 15–16
Urine sample collection form	A sample urine collection form to track urine specimens	Supplementary data page 17
Stool collection protocol for patients	A protocol that details how patients can self-collect stool samples and ship them to a receiving institution	Supplementary data pages 18–21
Stool sample collection form	A sample stool collection form to track urine specimens	Supplementary data page 22
Stone sample collection and processing for physicians	A protocol that details the collection and processing of kidney stone samples by physicians/investigators	Supplementary data pages 23–24
***Sample processing***		
Urine processing for investigators	A protocol that details storage, DNA extraction and sequencing of urine samples	Supplementary data pages 25–26
Stool processing for investigators	A protocol that details storage, DNA extraction and sequencing of stool samples	Supplementary data pages 27–29
***Data analysis***		
16S mapping file template	Template files with standardized variables for clinical data. The mapping file will be populated with data collected from patients	On server
Shotgun metadata template		On server

IRB, Institutional Review Board; MWAS, metagenome-wide association study.

### Sample collection

As with any clinical study, a power analysis should first be conducted to determine the sample size needed for the study objectives — statistical packages such as Evident^23^ and msWaldHMP^24^ have been developed specifically for power analysis of microbiome studies.

Several sample collection protocols were constructed and agreed upon that have been validated for previous microbiome studies. Consensus-agreed protocols include techniques for collection of mid-stream voided urine and stool samples, which are performed by the patients themselves, as well as protocols for stone collection and the collection of catheterized or upper urinary tract (UUT) urine, which are conducted by physicians (Table 1).

Within the urinary tract, different anatomical niches have distinct microbial communities^3,13^. These include the UUT niche, which can be sampled cystoscopically using an open-ended ureteral catheter placed up the ureter and in the renal pelvis or via a ureteroscope within the renal pelvis or once percutaneous kidney access is obtained, with urine aspirated using a sterile syringe^25^. Urine from this anatomical niche is needed to acquire microbes that are mechanistically involved in stone formation. Such bacteria, present in the direct vicinity of stone formation, can directly influence the lithogenic potential of metabolites or bind minerals together through the production of biofilms^26^. Downstream from the UUT, the bladder niche can be sampled by catheterization or suprapubic aspirate, the urethra can be sampled by a swab (distal urethra only) and the urinary meatus can be sampled using a swab.

In stone microbiome studies, study participants might be requested to provide a stool sample, a urine sample and/or a stone sample. Stool samples would be self-collected by study subjects using the stool collection kit and protocol (Supplementary information 1, page 18–21). We recommend that stool be collected using an in-commode collection system (such as Fisher Science Catalogue # 2544208) to ensure sampling of an adequate representation of the microbiome.

Urine samples (UUT, catheterized or mid-stream voided specimen) should be collected from all study participants, either in clinic, in the preoperative area or with an at-home kit according to the appropriate protocol (Supplementary information 1, page 11–14). For self-collected mid-stream voided urine, we recommend use of a Peezy Midstream urine collection device (Forte Medical) to reduce potential contamination. Although alternative devices are available, a 2019 study by Southworth et al.^27^ found that the Peezy system was associated with lower contamination than standard urine collection cups when used for urinary microbiome analyses.

The panel recommends that both stool and urine samples should be collected before the stone removal procedure and before preoperative or perioperative antibiotics (as appropriate) are administered (specifically to eliminate any false positives generated by the immediate use of antibiotics in patients with stones but not in control groups) or by the clinician during the procedure in the case of UUT or stone samples. Stone samples should be collected during the surgical procedure for removal (ureteroscopy or percutaneous nephrolithotomy), with a portion of the sample sent for clinical analysis of composition. A minimum of 500 mg of stone sample should be available for DNA extractions to provide sufficient biomass for downstream processing^3,13^. The consortium recommends that stone samples should be rinsed with sterile PBS to remove potential host bacteria contamination, flash frozen in liquid nitrogen and pulverized with a bullet blender^3,13^. The pulverized stone powder can then be used for DNA extraction.

The panel agreed that the hierarchy of urine quality for studies for scientific rigour would be ideally UUT urine, followed by catheterized or aspirated urine, then mid-stream voided urine. To understand the mechanistic microbiology of the urinary tract, sampling from UUT or catheterized urine must be performed if possible. If a researcher only has access to voided urine specimens — such as studies involving control patients in whom UUT or catheterized urine is not feasible to obtain — the researchers must be aware of the limitations associated with interpreting the mechanistic basis of these data. Mid-stream urine specimens are often not ‘clean’ and these specimens primarily sample the distal urethra with periurethral and other contaminants in urine specimens from men and distal urethral, periurethral and vulval contaminants in urine specimens from women^28–30^. The consortium recognizes that samples derived from different urinary anatomical niches between patients (for example, UUT in one patient with voided urine in another) would not provide an accurate comparison from a microbiota analytical perspective.

For nomenclature purposes, the consortium agreed that microbiome data from samples of voided urine should be referred to as ‘genitourinary microbiome’, data from catheterized urine samples should be known as ‘bladder urine microbiome’ and data from the UUT as ‘kidney urinary microbiome’.

### Storage, preservation and processing of samples

Urine and stone samples should be stored in a preservative (boric acid when culture is planned or AssayAssure when sequencing is planned^31^) at 4 °C before being put into storage at −80 °C within 24 h of collection. For urine samples, AssayAssure minimizes alterations in the microbial community if urine samples are to be stored at room temperature for longer than 1 h^31^; if urine samples are frozen within 1 h, AssayAssure is not needed. Of note, neither boric acid nor AssayAssure is recommended if downstream metabolomic assays or other clinical assays are planned, such as a 24-h urine collection for metabolic risk factors, as boric acid or other preservatives lead to substantial alterations of the urinary metabolites^31,32^. In these cases, the consortium recommends that an aliquot of urine is subsampled and placed into preservative for downstream microbiome analyses, whereas another sample without preservative is used for metabolomic analyses. All samples should be transferred on dry ice or shipped with ice packs in a polystyrene container and should be stored at −80 °C as soon as they are received at the laboratory. Repeated freezing and thawing of samples should be avoided, as it can affect microbial community composition by unevenly lysing bacteria in the sample followed by degradation of the DNA^33^.

For stool samples, the Faecal Aliquot Straw Technique will be performed on the specimens received^34^. This technique has been validated to both minimize changes to the microbiome prior to downstream analyses and provide suitable material for metaculturomics or in vivo studies. Stool samples must arrive at 4 °C within 24 h of collection with no evidence of freezing to prevent repeated freeze–thaw cycles, which can lead to bacterial lysis and alter the microbial community. Inside a biological hood, four sample straws should be inserted throughout the faecal sample, provided that there is enough faecal material; if not enough material is available, as many straws as possible should be inserted. Filled straws should then be snap-frozen and stored in sterile 15-ml tubes (two straws per tube) at −80 °C. The Faecal Aliquot Straw Technique has the benefit that, in addition to preserving samples for metagenomic or other omics analyses, the samples can also be used for subsequent in vitro or in vivo studies.

Protocols for sample collection and processing have been constructed **(**Supplementary information 1, Box 1). Protocols include clinical metadata definitions, a sample questionnaire to collect pertinent information from patients, collection of mid-stream urine, collection of UUT urine, collection of stool, processing of samples for DNA and templates for mapping files required for bioinformatic analysis.

### DNA extraction

To ensure consistent extraction of DNA from all samples, the consensus agreement was for use of an automated DNA extraction process, which reduces the amount of user bias and increases the consistency of microbiome data^35^. For all DNA extractions, negative controls that include sterile water and all extraction reagents should be included alongside every set of samples; positive controls should include a commercial standardized mixed microbial community sample that is run with every sequencing batch. Subsequently, the panel recommends that all extractions should be verified using gel electrophoresis and concentrations quantified using a Nanodrop Spectrophotometer or Qubit Fluorometer (Thermo Scientific).

We recommend that all samples should be sequenced. None of the negative controls from any preparation should have any quantifiable DNA, but they should be sequenced in parallel with positive samples to help to identify sequencing errors and possible contamination. Any taxa present at a non-zero abundance in positive controls that are not known members of the mock community should be removed from all samples as contaminants^36^. Low biomass samples — such as catheterized urine or stone samples — are recommended to be sequenced at least twice to ensure reproducibility^13^. Replicate samples should be compared using a dissimilarity index such as the phylogenetic UniFrac^37^ or non-phylogenetic Bray–Curtis^38^ to determine whether sequencing artifacts are present. For stone and urine samples, we recommend that only samples with a minimum of 2,000 reads but ideally >3,000 reads and that do not resemble negative controls, should be used for downstream analyses. For stool samples, only those with >10,000 reads should be used. These read thresholds have been empirically determined in past studies as being adequately representative of the diversity present in stone, urine and stool samples^3^.

### Sequencing and data analysis

The recommendation for sequencing is for paired-end sequencing, using either the V4 region of the 16S rRNA gene or shotgun metagenomic sequencing on an Illumina MiSeq or HiSeq, respectively; for the latter, Illumina’s NextSeq or Novaseq are also possibilities. The Illumina platforms produce longer and more accurate reads, with higher throughput than other contemporary platforms and are those most widely used for microbiome studies^39^. If these platforms are not available, other sequencing platforms can be used, as the choice of primer has a greater effect on downstream data than the specific sequencing platform^40^.

**Fig. 1:**
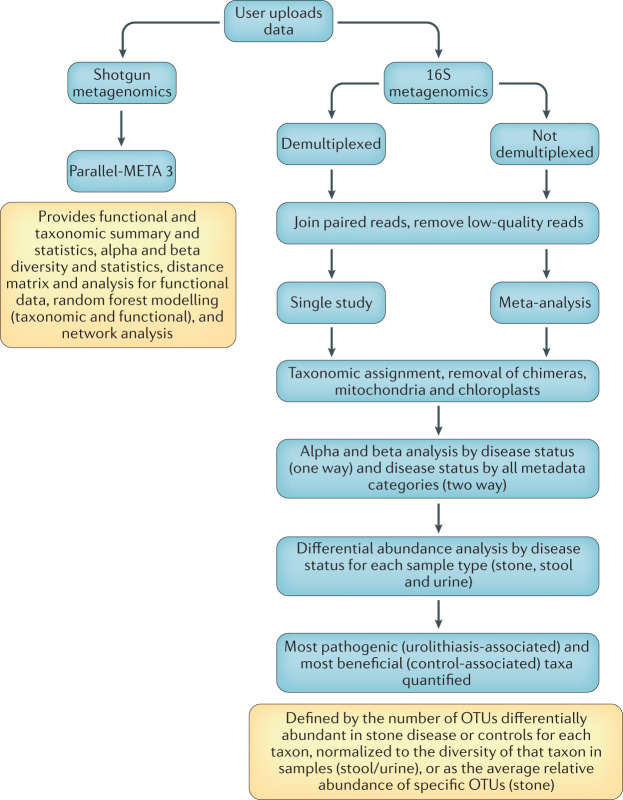
Microbiome data analytical workflow. The flowchart illustrates the analytical steps following data upload for both shotgun and 16S studies, which is automated on upload to the secure analytical server. OTUs, operational taxonomic units.

An automated analytical pipeline, which is a freely available resource (Box 1), was developed to analyse urolithiasis MWAS in a consistent manner. The automated pipeline is capable of processing both 16S rRNA and shotgun metagenomic data. With this pipeline, the user uploads raw sequencing data and a mapping file with clinical metadata as input, and then processes the data to generate microbial profiles for each sample. The pipeline performs statistical comparisons to determine which taxa (16S rRNA) or genes (shotgun metagenomics) associate positively or negatively with urolithiasis in one-way and two-way analyses to determine which clinical metadata associate with the microbiome in a way that affects urolithiasis. The pipeline analyses new datasets individually, and then incorporates the new dataset with all previous datasets to produce updated meta-analyses as new data are acquired (Fig. 1, Box 2). Multiple analytical programmes have been developed to handle high-throughput 16S rRNA data, including QIIME^41^, Mothur^42^ and DADA2 (ref.^43^). Several scripts from these pipelines are employed in our automated pipeline. To summarize the analytical steps in the pipeline (Fig. 1), paired end reads from each study being analysed are joined using fastq-join^44^; the reads are quality controlled, trimmed and demultiplexed using default parameters in QIIME 1.9.1 (ref.^45^). After demultiplexing, reads are assigned to amplicon sequence variants (ASVs) in DADA2 using an open-reference strategy, for which the Silva 138 SSURef and NCBI databases^46^ were used as the initial references for mapping. Reads that do not match the reference database are subsequently clustered de novo and representative sequences from each cluster are used for classification. Low-abundance ASVs (<10 reads in a single dataset), chimeras and ASVs classified as mitochondria or chloroplasts are removed from further analysis^3^. The DECONTAM algorithm is used to remove contamination from samples^47,48^. Datasets are merged using the merge_otu_tables.py script in QIIME and the merged tables are normalized using the DESeq2 normalization protocol, which corrects for sequencing depth and composition bias across samples^49^.

Alpha and beta diversity are calculated using the phylogenetic metrics, PD_whole_tree and weighted UniFrac distance matrices^50,51^. Differential abundance of ASVs between individuals within comparison study populations are assessed using the DESeq2 algorithm^49^. To determine the most dysregulated taxa, significantly different ASVs are reduced to the lowest assigned taxonomy and the number of significantly different ASVs assigned to those taxa are normalized to the total number of ASVs assigned to those taxa in the whole dataset. The values normalized to taxon diversity are ranked as more health-associated for those taxa with higher values in the control populations, or more urolithiasis-associated for those taxa with higher values in the stone disease population^52^. For the stone-associated microbiome, which is less diverse than the gut and urinary tract microbiome and will not have a control population for comparison, the most potentially relevant taxa are determined by ranking each ASV by the mean relative abundance across all samples in the dataset.

For shotgun metagenomic datasets, data are processed on the analytical server through the parallel-meta3 pipeline, which provides a broad set of analyses that includes 16S rRNA gene sequence extraction for alpha and beta diversity analyses, as well as functional profiling and network analysis at multiple levels^53^.

Box 2 The MICROCOSM server interface Once logged into the secure online server, users have access to instructions for use, all consensus-agreed protocols, mapping templates required for 16S and shotgun metagenomics sequencing, raw data and results files from all previous metagenome-wide association studies to enable data sharing, and a means of uploading new data. The MICROCOSM server homepage is split into a number of different sections:

Instructions

- Includes instructions on how to conduct microbiome studies and use the server
- Also covers information on downloading protocols, mapping templates and results, as well as conducting a microbiome clinical study, how to perform sequencing and analysis, and how to upload data

Protocols

- Stool, urine and kidney stone collection and storage
- DNA extraction sequencing
- Metaculturomic analysis
- Questionnaires for use in clinical studies

Mapping templates

- Includes one mapping template for 16S rRNA and one for shotgun metagenomics, each of which requires different formatting
- Currently, the shotgun metagenomics pipeline is set up only to look at associations with urolithiasis (not metadata)

Uploaded data files

- Sequencing files will include a forward, reverse and barcode file name of the mapping file, as shown in Supplementary information 1, or a folder of demultiplexed reads
- Upon uploading data, the user will be asked the name of the files or directory, the name of the mapping file and whether the data are 16S or shotgun

Results

- Results will be presented in a series of directories
- There will be one directory per study and an additional directory for the meta-analysis of all 16S studies combined

### Metaculturomics

In order to cross-validate sequencing results, to ensure that the detected bacteria come from the stones rather than the surrounding urine, and to isolate bacteria for mechanistic studies, the consortium recommends that a metaculturomic approach be used (Table 2). An expanded quantitative urine culture protocol has been developed for urine and stone samples^13^, whereas other culturomic approaches have been developed for stool samples^54^. For the expanded quantitative urine culture protocol, large volumes of sample (10 µl and 100 µl) are plated onto a variety of media, including blood agar, chocolate agar, colistin and nalidixic acid agar, and CDC anaerobe blood agar. These plates are incubated under multiple atmospheric conditions (aerobic, anaerobic, 5% CO_2_) for 48 h; the minimum detection threshold is 10–100 colony-forming units per millilitre^13^. For stool samples, which contain greater taxonomic and metabolic diversity than urine and stone samples^3^, a wider variety of conditions are needed to adequately capture the diversity present. The most successful culture conditions relative to capturing broad diversity include aerobic and anaerobic culturing in blood agar with or without rumen fluid, marine broth, or trypticase soy broth^54^. Preincubation of stool or urine samples in broth, using the conditions defined (Table 2), often increases the diversity of bacteria isolated, as preincubation will put all bacteria in the sample in an active metabolic state^13,54^. Notably, specific taxa or metabolic functions can be targeted by using specialized media types and/or conditions and might be necessary for additional mechanistic studies. For instance, using media with oxalate as a sole carbon and energy source, investigators previously isolated *Oxalobacter formigenes*, which is a bacterium that helps to reduce the amount of dietary oxalate that is absorbed into the blood stream^55^. Other metabolomic analyses have pointed to specific urinary metabolites linked to *Lactobacillus* spp. that are negatively associated with urolithiasis^3^. Studies designed to mechanistically verify the effect of these metabolites on lithogenesis, along with the source of these metabolites, are needed.

**Table 2:** Culturomic conditions for the isolation of bacteria from either urine and kidney stones^13^ or stool^54^

Media	Temperature	Atmosphere	Pre-incubation	Incubation period
***Urine/kidney stone***				
Blood agar	37 °C	Aerobic	No	48 h
Blood agar + colistin and nalidixic acid	37 °C	Anaerobic	No	48 h
CDC anaerobe blood agar	37 °C	5% CO_2_	No	48 h
Chocolate agar	37 °C	5% CO_2_	No	48 h
***Stool***				
Blood agar	37 °C	Aerobic	Yes	Up to 30 days
Blood agar + rumen fluid	37 °C	Anaerobic	Yes	Up to 30 days
Blood agar + marine broth	37 °C	Microaerophilic	Yes	Up to 30 days
Blood agar + trypticase soy broth	37 °C	Microaerophilic	Yes	Up to 30 days

To isolate bacteria, morphologically distinct colonies on agar plates should be streaked onto fresh plates of the same media for isolation and subsequently preserved in 10–15% glycerol at −80 °C for future identification via sequencing or matrix-assisted laser desorption and/or ionization time-of-flight and mechanistic studies.

### Development of a centralized repository

All consensus protocols, templates for metadata files, instructions for conducting MWAS, raw sequencing data for each study, as well as result files for individual studies and meta-analyses were placed in a secure, encrypted server (Box 1, Supplementary information 1). Importantly, although all protocols have been validated and standardized by the MICROCOSM consortium, all clinical microbiome studies performed according to these protocols must acquire institutional review board approval at the researchers’ own institution before beginning the study.

## Future directions

Numerous biological and experimental factors are known to affect microbiome composition, such as ethnicity, diet, sex and pharmaceuticals^56^. Additionally, the experimental approach in metagenomic studies is well accepted to have a large effect on the downstream data and interpretation^21^. Sample collection, preservation and storage, DNA extraction, library preparation and DNA contamination can all affect taxonomic and functional compositions of metagenomes^57–64^. Thus, concern for potential experimental bias provides the driving rationale for the need to standardize experimental approaches to the greatest extent possible in order to determine between experimental and biological and/or environmental factors that affect microbiome composition in an urolithiasis-specific manner.

The development of microbiome-based actionable therapies to prevent the onset and recurrence of urinary stones will depend on confident assessment of environmental factors that affect both the microbiome and urolithiasis. Thus, differentiating between experimental and biological biases in microbiome studies associated with stone disease will require standardization of experimental protocols across the field. In turn, standardization will enable comparison of results between studies and expand the number of MWAS that can be performed by investigators who did not previously have the capability to perform MWAS. The findings of this consensus agreement provide a much needed framework with which to work towards this crucial goal. Notably, however, standardization of sample collection, storage, DNA extraction, library preparation and analytical protocols do not affect study-specific objectives, unless the study is aimed at comparing different aspects of a protocol specifically, as all MWAS must perform the experimental steps standardized in this Consensus Statement.

To move past associative studies and understand the mechanistic basis for the microbiome contributions to urolithiasis, the isolation of bacteria from stool, urine or stones must be performed. Metaculturomics, which employs a wide range of media types and culture conditions to broadly capture the diversity of bacteria present in a sample, has proven to be an effective means of recapitulating the diversity present in human-derived samples^13,54^. Such approaches have been used to successfully culture numerous bacterial species that were previously undetected or considered to be uncultivable, such as species from the *Erysipelatoclostridium*, *Dielma* and *Butyricicoccus* genera, among others^65^, and are, therefore, an effective means of acquiring bacteria perceived to be important through culture-independent studies.

This consensus agreement and the recommendations of the panel will be particularly useful for investigators who do not currently have the necessary equipment or expertise to carry out this type of research, as the protocols offer a means of consistently collecting samples and shipping them to laboratories equipped to conduct MWAS, and a guide to those interested in microbiome research in other areas of urology.

## Conclusions

This Consensus Statement describes the development of the first international multi-institutional consortium for microbiome in urinary stone research (MICROCOSM) and formulation of a consensus agreement that provides a standardized, validated approach with clear protocols for conducting this research, as well as development of a robust analytical platform that will be widely and freely available using our unique secure online server. This work sets the benchmark for the field and provides a future resource for further microbiome-based studies, as well as facilitating multi-institution collaboration in advancing research of the microbiome in stone disease.

## Supplementary information

Supplementary Information

## Glossary

Metaculturomics: A technique defined by the use of multiple culture conditions and media types to maximize the diversity of bacteria isolated from a particular environment such as host-associated stool or urine.
16S rRNA gene sequencing: The 16S rRNA gene is a conserved microbial gene with hypervariable regions that is conventionally used for the taxonomic classification of bacteria and archaea. When targeted 16S rRNA gene sequencing is conducted through high-throughput sequencing methodologies, a microbial profile of a sample can be obtained and compared with other samples.
Shotgun metagenomic sequencing: The metagenome of a host-associated microbiota contains all of the microbial genes present in the community. Shotgun metagenomic sequencing refers to the fragmentation and sequencing of the metagenome from a sample. Fragmented sequences can be bioinformatically assembled after sequencing to obtain a complete picture of a sample’s metagenome.
Dissimilarity index: A statistical method used in ecology to quantify differences in community composition based on multivariate count data.
Unifrac: A dissimilarity index that incorporates the phylogenetic relatedness of bacteria in a sample when determining differences between microbiome samples. Both unweighted measures, which quantify the presence or absence of bacteria, and weighted measures, which integrate relative abundances of bacteria, are available.
Bray–Curtis: A non-phylogenetic dissimilarity index that is solely based on counts of individual taxa within a sample.
Amplicon sequence variant: A unit of taxonomic classification based on 16S rRNA sequences recovered from high-throughput sequencing assays. Amplicon sequence variants are defined through single-nucleotide changes in comparison with well-annotated 16S rRNA databases and are thought to be a more precise and reproducible means of classifying bacteria and archaea than conventional means.

## References

[1] McDonald D (2018). American Gut: an open platform for citizen science microbiome research. mSystems.

[2] Ticinesi A (2018). Understanding the gut–kidney axis in nephrolithiasis: an analysis of the gut microbiota composition and functionality of stone formers. Gut.

[3] Zampini A, Nguyen AH, Rose E, Monga M, Miller AW (2019). Defining dysbiosis in patients with urolithiasis. Sci. Rep..

[4] Chen Y-Y (2019). Microbiome–metabolome reveals the contribution of gut–kidney axis on kidney disease. J. Transl Med..

[5] Tao S (2019). Understanding the gut–kidney axis among biopsy-proven diabetic nephropathy, type 2 diabetes mellitus and healthy controls: an analysis of the gut microbiota composition. Acta Diabetol..

[6] Chewcharat A, Curhan G (2021). Trends in the prevalence of kidney stones in the United States from 2007 to 2016. Urolithiasis.

[7] Tasian GE (2016). Annual incidence of nephrolithiasis among children and adults in South Carolina from 1997 to 2012. Clin. J. Am. Soc. Nephrol..

[8] Saigal CS, Joyce G, Timilsina AR, The Urologic Diseases in America Project. (2005). Direct and indirect costs of nephrolithiasis in an employed population: opportunity for disease management?. Kidney Int..

[9] Alelign T, Petros B (2018). Kidney stone disease: an update on current concepts. Adv. Urol..

[10] Cochat P, Rumsby G (2013). Primary hyperoxaluria. N. Engl. J. Med..

[11] Giannossi, M. L. & Summa, V. in *An Introduction to the Study of Mineralogy* 123–147 (InTech, 2012).

[12] Schultz, L. N., Connolly, J., Lauchnor, E., Hobbs, T. A. & Gerlach, R. in *The Role of Bacteria in Urology* 41–49 (Springer, 2016).

[13] Dornbier RA (2019). The microbiome of calcium-based urinary stones. Urolithiasis.

[14] Stern JM (2016). Evidence for a distinct gut microbiome in kidney stone formers compared to non-stone formers. Urolithiasis.

[15] Miller AW, Choy D, Penniston KL, Lange D (2019). Inhibition of urinary stone disease by a multi-species bacterial network ensures healthy oxalate homeostasis. Kidney Int..

[16] Tang R (2018). 16S rRNA gene sequencing reveals altered composition of gut microbiota in individuals with kidney stones. Urolithiasis.

[17] Suryavanshi MV (2016). Hyperoxaluria leads to dysbiosis and drives selective enrichment of oxalate metabolizing bacterial species in recurrent kidney stone endures. Sci. Rep..

[18] Barr-Beare E (2015). The interaction between Enterobacteriaceae and calcium oxalate deposits. PLoS ONE.

[19] Xie J (2020). Profiling the urinary microbiome in men with calcium-based kidney stones. BMC Microbiol..

[20] Batagello CA, Monga M, Miller AW (2018). Calcium oxalate urolithiasis: a case of missing microbes?. J. Endourol..

[21] Nayfach S, Pollard KS (2016). Toward accurate and quantitative comparative metagenomics. Cell.

[22] Gratzke C (2015). EAU guidelines on the assessment of non-neurogenic male lower urinary tract symptoms including benign prostatic obstruction. Eur. Urol..

[23] Vázquez-Baeza Y, Pirrung M, Gonzalez A, Knight R (2013). EMPeror: a tool for visualizing high-throughput microbial community data. GigaScience.

[24] Mattiello F (2016). A web application for sample size and power calculation in case-control microbiome studies. Bioinformatics.

[25] Bier S (2018). Performance of urinary markers for detection of upper tract urothelial carcinoma: is upper tract urine more accurate than urine from the bladder?. Dis. Markers.

[26] Hobbs T, Schultz LN, Lauchnor EG, Gerlach R, Lange D (2018). Evaluation of biofilm induced urinary infection stone formation in a novel laboratory model system. J. Urol..

[27] Southworth E (2019). A cross-sectional pilot cohort study comparing standard urine collection to the Peezy midstream device for research studies involving women. Female Pelvic Med. Reconstr. Surg..

[28] Bajic P (2018). Male bladder microbiome relates to lower urinary tract symptoms. Eur. Urol. Focus.

[29] Wolfe AJ (2012). Evidence of uncultivated bacteria in the adult female bladder. J. Clin. Microbiol..

[30] Chen YB (2020). The urethral microbiota–a missing link in the female urinary microbiota. J. Urol..

[31] Jung CE (2019). Benchmarking urine storage and collection conditions for evaluating the female urinary microbiome. Sci. Rep..

[32] Wang X (2019). Influence of storage conditions and preservatives on metabolite fingerprints in urine. Metabolites.

[33] Gorzelak MA (2015). Methods for improving human gut microbiome data by reducing variability through sample processing and storage of stool. PLoS ONE.

[34] Romano KA (2018). Fecal aliquot straw technique (FAST) allows for easy and reproducible subsampling: assessing interpersonal variation in trimethylamine-N-oxide (TMAO) accumulation. Microbiome.

[35] Song E-J, Lee E-S, Nam Y-D (2018). Progress of analytical tools and techniques for human gut microbiome research. J. Microbiol..

[36] Karstens L (2019). Controlling for contaminants in low-biomass 16S rRNA gene sequencing experiments. mSystems.

[37] Lozupone C, Lladser ME, Knights D, Stombaugh J, Knight R (2011). UniFrac: an effective distance metric for microbial community comparison. ISME J..

[38] Beals EW (1984). Bray-Curtis ordination: an effective strategy for analysis of multivariate ecological data. Adv. Ecol. Res..

[39] Pollock J, Glendinning L, Wisedchanwet T, Watson M (2018). The madness of microbiome: attempting to find consensus “best practice” for 16S microbiome studies. Appl. Environ. Microbiol..

[40] Tremblay J (2015). Primer and platform effects on 16S rRNA tag sequencing. Front. Microbiol..

[41] Navas-Molina, J. A. et al. in *Methods in Enzymology* Vol. 531 371–444 (Elsevier, 2013). 10.1016/B978-0-12-407863-5.00019-8 PMC4517945 24060131

[42] Hiltemann SD (2019). Galaxy mothur Toolset (GmT): a user-friendly application for 16S rRNA gene sequencing analysis using mothur. GigaScience.

[43] Callahan BJ (2016). DADA2: high-resolution sample inference from Illumina amplicon data. Nat. Methods.

[44] Aronesty E (2013). Comparison of sequencing utility programs. Open Bioinform. J..

[45] Caporaso JG (2012). Ultra-high-throughput microbial community analysis on the Illumina HiSeq and MiSeq platforms. ISME J..

[46] Balvo M, Huson DH (2017). SILVA, RDP, Greengenes, NCBI and OTT — how do these taxonomies compare?. BMC Genomics.

[47] Davis NM, Proctor DM, Holmes SP, Relman DA, Callahan BJ (2018). Simple statistical identification and removal of contaminant sequences in marker-gene and metagenomics data. Microbiome.

[48] Karstens L (2019). Controlling for contaminants in low-biomass 16S rRNA gene sequencing experiments. mSystems.

[49] Love MI, Huber W, Anders S (2014). Moderated estimation of fold change and dispersion for RNA-seq data with DESeq2. Genome Biol..

[50] Caporaso JG (2010). QIIME allows analysis of high-throughput community sequencing data. Nat. Methods.

[51] Lozupone C, Hamady M, Knight R (2006). UniFrac–an online tool for comparing microbial community diversity in a phylogenetic context. BMC Bioinforma..

[52] Wilkins LJ, Monga M, Miller AW (2019). Defining dysbiosis for a cluster of chronic diseases. Sci. Rep..

[53] Jing G (2017). Parallel-META 3: comprehensive taxonomical and functional analysis platform for efficient comparison of microbial communities. Sci. Rep..

[54] Lagier J-C (2016). Culture of previously uncultured members of the human gut microbiota by culturomics. Nat. Microbiol..

[55] Allison MJ, Dawson KA, Mayberry WR, Foss JG (1985). Oxalobacter formigenes gen. nov., sp. nov.: oxalate-degrading anaerobes that inhabit the gastrointestinal tract. Arch. Microbiol..

[56] Zhernakova A (2016). Population-based metagenomics analysis reveals markers for gut microbiome composition and diversity. Science.

[57] Voigt AY (2015). Temporal and technical variability of human gut metagenomes. Genome Biol..

[58] Sinha R, Abnet CC, White O, Knight R, Huttenhower C (2015). The microbiome quality control project: baseline study design and future directions. Genome Biol..

[59] Kennedy NA (2014). The impact of different DNA extraction kits and laboratories upon the assessment of human gut microbiota composition by 16S rRNA gene sequencing. PloS ONE.

[60] Jones MB (2015). Library preparation methodology can influence genomic and functional predictions in human microbiome research. Proc. Natl Acad. Sci. USA.

[61] Ames SK (2015). Using populations of human and microbial genomes for organism detection in metagenomes. Genome Res..

[62] Tanner MA, Goebel BM, Dojka MA, Pace NR (1998). Specific ribosomal DNA sequences from diverse environmental settings correlate with experimental contaminants. Appl. Environ. Microbiol..

[63] Salter SJ (2014). Reagent and laboratory contamination can critically impact sequence-based microbiome analyses. BMC Biol..

[64] Weiss, S. J. et al. Effects of library size variance, sparsity, and compositionality on the analysis of microbiome data. Report No. 2167-9843 (PeerJ PrePrints, 2015).

[65] Zou Y (2019). 1,520 reference genomes from cultivated human gut bacteria enable functional microbiome analyses. Nat. Biotechnol..

