# Supplementary material for: Standardization of microbiome studies for urolithiasis: an international consensus agreement
Source: Nat Rev Urol. 2021 Mar 29;18(5):303–11. doi: 10.1038/s41585-021-00450-8 (PMC8105166; doi:10.1038/s41585-021-00450-8)
Supplement: Supplementary file 1 — Supplementary Information [file 41585_2021_450_MOESM1_ESM.pdf]

nature portfolio

https://doi.org/10.1038/s41585-021-00450-8

Supplementary information

Standardization of microbiome studies for urolithiasis: an international consensus agreement

In the format provided by the authors and unedited

Standardization of Microbiome Studies for Urolithiasis: An International Consensus Agreement

Supplemental Data

If you use the server or protocols described here, this consensus statement must be cited.

1. Instructions for MWAS and Automated analytical server

MICROCOSM: Microbiome contributions on the complexity of the stone matrix

Objectives: The objectives of MICROCOSM are to standardize the protocols for microbiome research within the urolithiasis field to minimize the technical biases and barriers associated with microbiome research, while allowing for the flexibility of study-specific objectives. In this way, MICROCOSM seeks to provide a robust foundation for multi-institutional studies and to be able to compare results across multiple independent studies.

*Important note: All clinical microbiome studies will require IRB approval at the institutional level. Protocols provided by MICROCOSM can be and has been used for IRB approvals.

Protocols available:

1. Sample collections & processing
2. Questionnaires for metadata collection
3. Mapping templates for metadata collation
4. Technical details for 16S & shotgun metagenomic sequencing
5. Automated bioinformatic pipeline for 16S and shotgun metagenomic sequencing that provides study specific and meta-analysis results with each new dataset added to the server
6. Metaculturomic analysis of stone & urine microbes

Sample collections & processing

Several sample collection protocols are present on the server that have been validated for microbiome studies. These include mid-stream voided urine protocols and stool collection protocols conducted by patients, as well as protocols for stone collection, or the collection of catheterized or upper urinary tract urine to be conducted by physicians.

For stool, there are two protocols needed:

1. Stool collection protocol for patients
2. Stool processing for investigators

For urine, there are two protocols needed:

1. Mid-stream voided urine sample collection protocol for patients
2. Urine processing for investigators

For stones, there is one protocol needed:

1. Stone sample collection & processing protocol for physicians & investigators

Automated data analysis

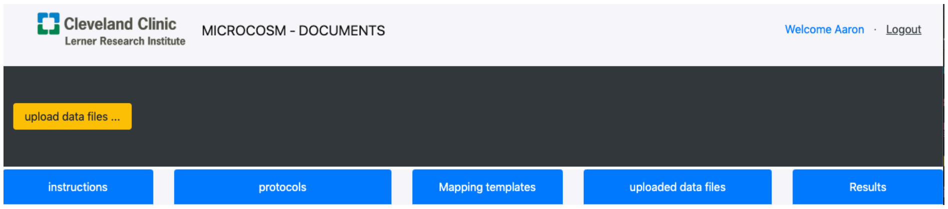

1. Automated data analysis of each study's data, along with incorporation of the dataset into a meta-analysis of past clinical microbiome studies of urolithiasis, is done at https://www.lerner.ccf.org/cms/miller/uscd/app/. Users will have to request an invitation to the server prior to uploading data.

2. Once logged in, users will have access to these instructions, a list of protocols as stated above, mapping templates required for 16S and shotgun metagenomic sequencing, results files, and a means to upload new data.

3. For 16S rRNA sequencing data, users will be provided data by their sequencing facility in one of two formats. First, data will not be demultiplexed. In this case, users will have 4 files: forward_reads.fastq, reverse_reads.fastq, barcode_reads.fastq, and mapping_file.txt. Please ensure that the mapping file is formatted according to the 16S_mapping_template.txt file on the server (under templates). User will have to enter in all associated metadata for each sample. Second, data may already be demultiplexed (sequence reads already assigned to samples). In this case, users will have a directory of read files (one for each sample) and a mapping file. Again, mapping files should be formatted appropriately.

4. For shotgun metagenomic data, users will be provided with a forward and reverse read file for each sample. Users should put these files into their own directory.

5. In any of the 3 cases above, users should compress all files together (create a .zip file) and upload the data to the server. Upon submission, data processing will commence. Completion time will depend on the number of samples and whether 16S or shotgun metagenomic sequencing was employed. Results are typically available within 2 days of submission.

Interpretation of results – Shotgun metagenomics

1. Currently, the shotgun metagenomics pipeline is run through parallel-meta3 and conducts a thorough analysis of associations in the data based on the status of urolithiasis as a grouping factor. Result output includes taxonomic and functional profiling by group (relative abundance, diversity, and cluster analysis), random Forest modeling to determine the most defining functions and taxonomic classes between groups, and microbe-microbe network analysis. In the future, more specific gene level analyses may be added along with the ability to conduct meta-analyses of shotgun metagenomic data.

Interpretation of results – 16S metagenomics

1. The 16S metagenomic pipeline is designed to identify significant associations between status of urolithiasis and all metadata categories in 2-way grouping factors. Results include diversity analyses (alpha and beta) for all 2-way comparisons, differential abundance analysis, and quantification of those taxa that most separate controls from cases in the stool and urine, as well as the most abundant taxa in the stone matrix. Additional 1-way analyses are provided for stone samples without healthy controls.

2. For diversity analyses (alpha and beta), file name will include the type of analysis, sample type, and statistical factors (i.e. alpha_stool_Group_Study_location.pdf, where Group=status of urolithiasis). P-values will be displayed in the top right corner of the graph, where Var1 = the p-value for the first variable (Group in the below example), Var2 = the p-value for the second variable (Study_location), and Var1_Var2 = the p-value for the interaction between the two factors.

Example of generated alpha diversity analysis:

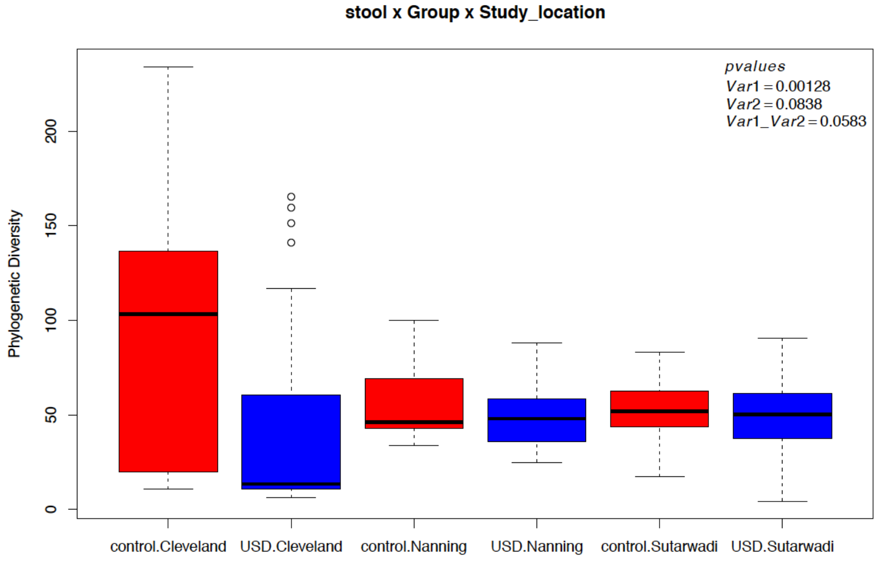

Example of generated beta diversity analysis

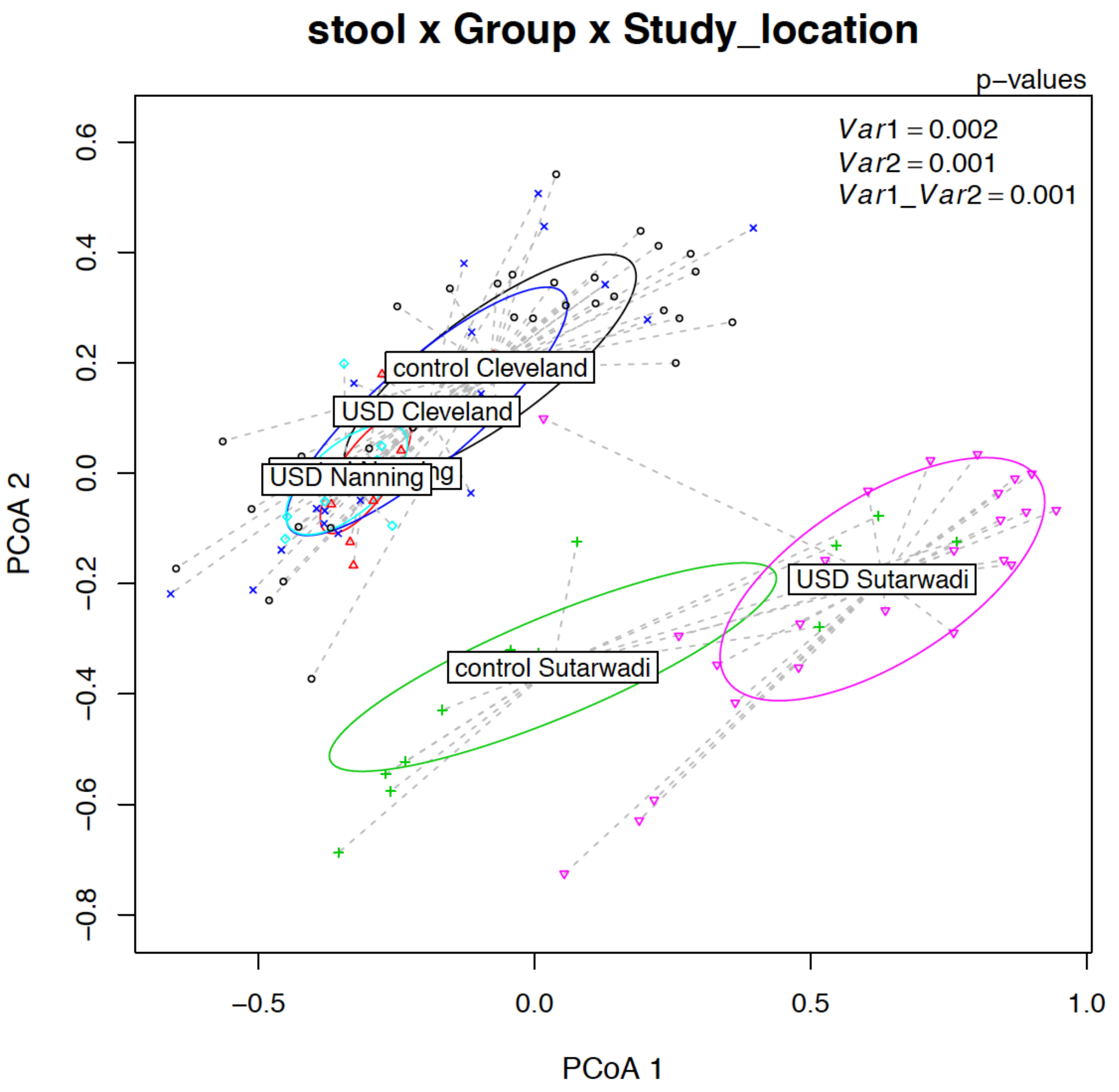

3. For the taxa that most separate cases from controls or is most abundant in stones, heatmaps will be generated as below, where taxa are listed as most associated with urolithiasis or most associated with healthy controls. These are quantified as the number of individual operational taxonomic units (OTUs) per taxa that are significantly enriched in one group or the other, normalized to the overall diversity of that taxon.

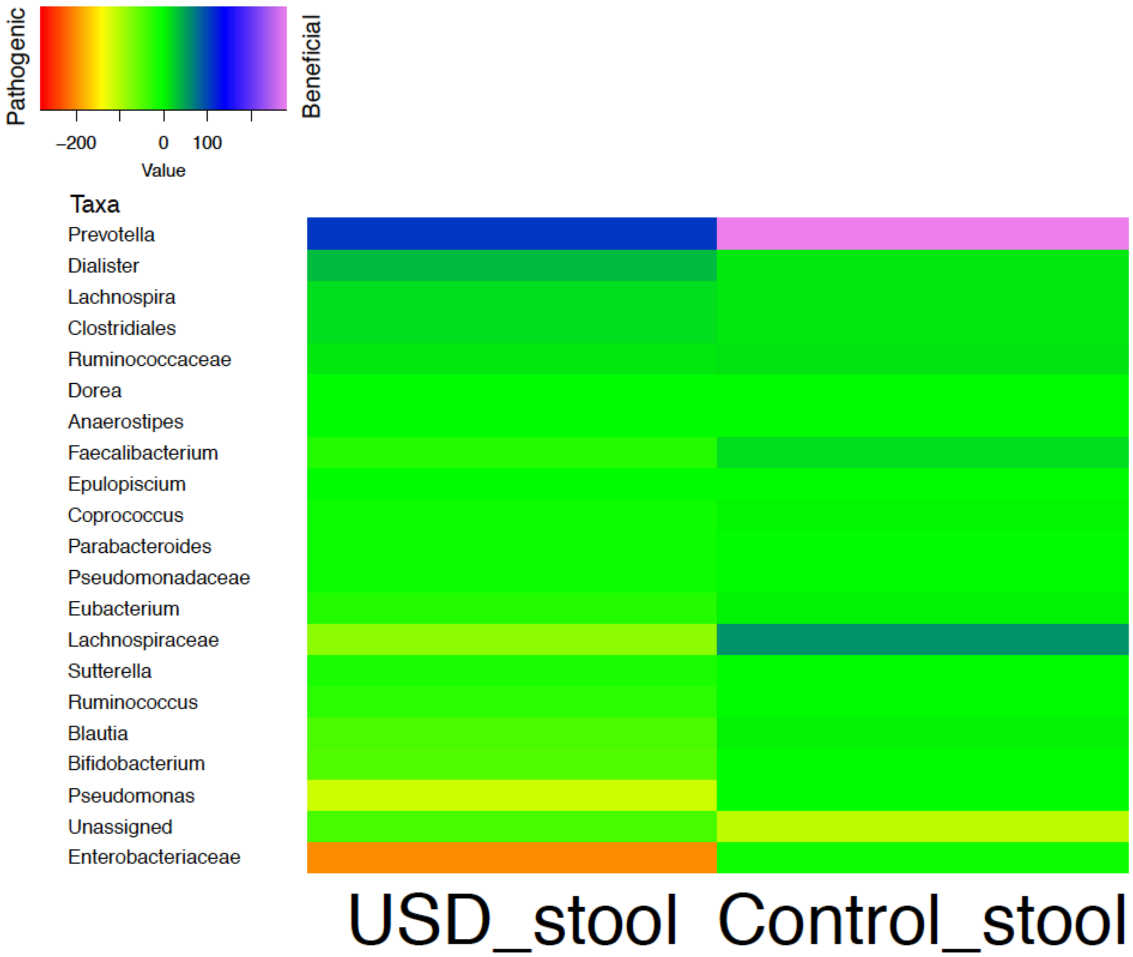

4. For the most abundant taxa in stones, the relative abundance of individual OTUs are plotted on a heatmap as below.

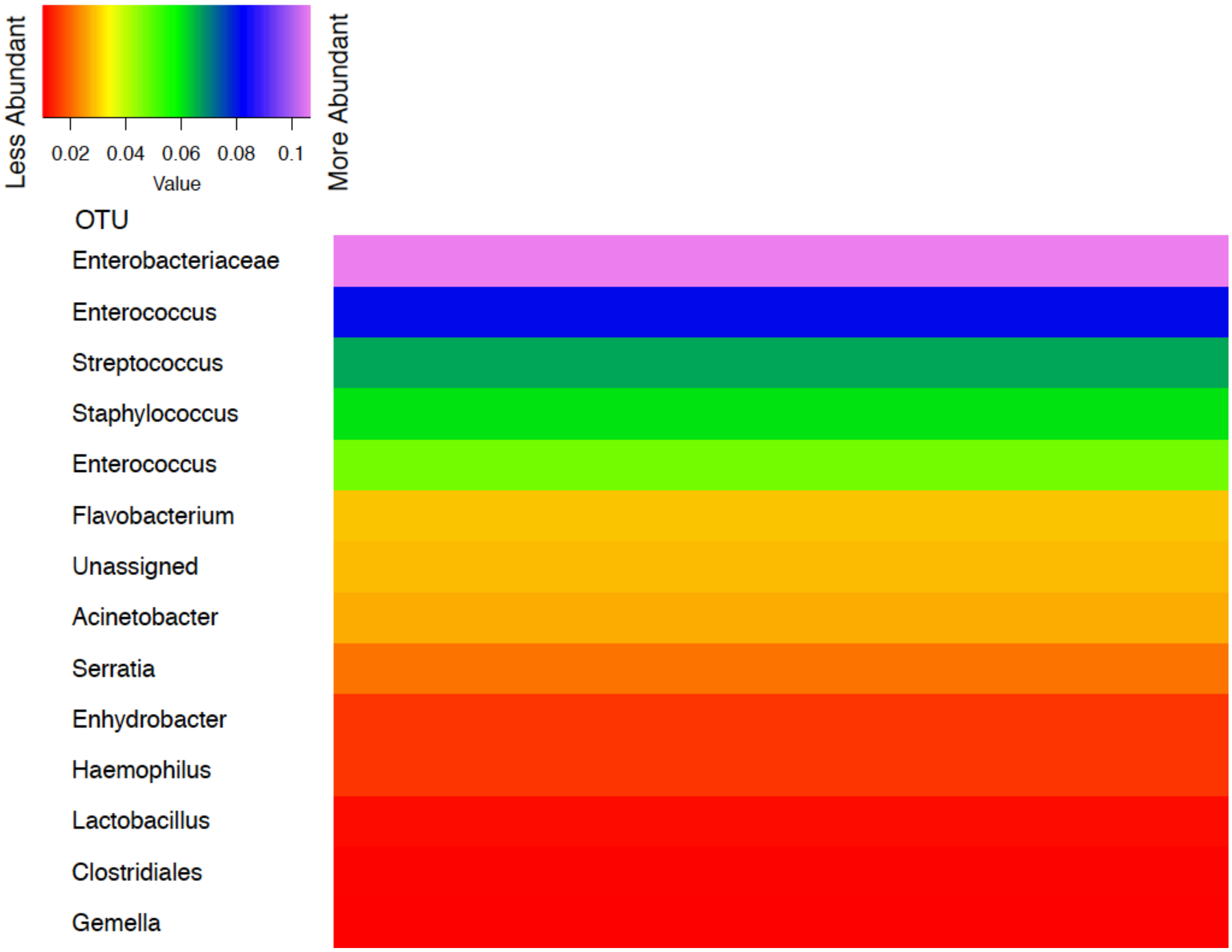

5. After data analysis is complete, users will be able to download the results for their individual studies as well as the results from the meta-analysis with their datasets now added.

Table S1. Metadata Definitions for 16S and shotgun studies

**Sample_Variables**	
#SampleID	Each entry in this list must be unique. Entries should be formatted as follows "6-digit identifier"_"Sample_type"_"Timepoint". As an example "665473_stool_1st_tri" is participant 665473, stool sample, and from the first tri after baseline. It is important to not use any spaces in the entry here.
BarcodeSequence	Generated as part of sequencing
LinkerPrimerSequence	Generated as part of sequencing
Description	Anonymous 6-digit identifier - Choose from pre-established list. Each patient gets one id even if they provide samples at multiple timepoints
Study	Descriptor for the study originators. Use the format of last name of first author, followed by year study started (i.e. Miller_2017)
Study_city	The city where participants were recruited
Study_country	The country where participants were recruited
Timepoint	Use "Baseline", "1st_tri", "2nd_tri", "After_abx", "2_weeks_after_abx", "1_tri_after_abx" case sensitive. Tri's are defined by 4 months after baseline and can be extended indefinitely. If participants take antibiotics, they will switch to the abx time line (1st tri after abx, etc.). These can also be extended indefinitely.
Sample_type	Use one of the following "peezy_urine", "stool", or "stone". These are case sensitive and other sample types can easily be added. Also source for urine 'UUT_urine', 'cathed_urine', 'voided_urine'
**Patient_Variables**	
Group	Use either "control", "stone", "recurrent_UTI" again case sensitive.
Age	Numerical age of patient
Age_group	Age group. Use one of the following "_18", "18_35", "35_50", "50_65", "65_". _18 is less than 18, 65_ is over 65, _ in other terms is "between".
Sex	Use either "Male" or "Female", case sensitive
Ethnicity	Use either of the following "Hispanic", "Native American_American indian", "Asian", "Black_African-american", "White", case sensitive
Education	Highest level of schooling completed "high school", "college", "associate degree", "bachelor's degree", "master's degree", "doctoral_professional degree"
Occupation	Use "employed", "self-employed", "not employed", "student" , "retired"
Zipcode	Numerical input
Height_inches	Numerical height of participant in inches
Height_cm	Numerical height of participant in cm
Weight_lb	Numerical weight of participant in pounds

Weight_kg	Numerical weight of participant in kilograms
BMI	Calculated from Height and weight
**Stone_Variables**	
Hx_stone	Use "no_history", "single_event", or "recurrent"
Year_1st_stone_episode	Free response - Question "what year was your first stone episode"
Stone_comp_predominant	Use one of the following "None", "Caox _Unk", "Caox _Mono", "Caox _Di", Caox_Caphos", "Caphos", "Uric_acid", "Caox_Uricacid", "Cystine", "Struvite". Case sensitive. Option for percentage
Stone_comp_other	Use one of the following "None", "Caox _Unk", "Caox _Mono", "Caox _Di", Caox_Caphos", "Caphos", "Uric_acid", "Caox_Uricacid", "Cystine", "Struvite". Case sensitive. Option for percentage. May be multiple other options
Stone_procedures	SWL "0", "1", "2_". URS "0", "1", "2_". PCNL "0", "1", "2_"
Stone_number_of_events	Use "0", "1", "2_"
Stone_location_right	use "kidney", "ureter" or "both", “NA”
Stone_location_left	use "kidney", "ureter" or "both", “NA”
No_of_stones_right	Numerical value for number of stones in right kidney
No_of_stones_left	Numerical value for number of stones in left kidney
Stone_largest_diameter_right	use "_5mm", "5-10mm", ">_10-20mm", "20mm_", “NA”
Stone_largest_diameter_left	use "_5mm", "5-10mm", ">_10-20mm", "20mm_", “NA”
Imaging_modality	use "Xray", "US", "CT"
Fam_hx_stone	Family history of USD. Use "yes", or "no", case sensitive.
Which_member	Which family member had stones.
**Antibiotic_Variables**	
antibiotic_12m	Antibiotic use in the last 12 months. Use "yes" or "no", case sensitive.
antibiotic_30d	Antibiotic use in the last 30 days. Use "yes" or "no", case sensitive.
antibiotics	Free response - Question "list all the antibiotics you have taken in the last 12 months"
**Past_Medical_History**	
Hx_Urinary_tract_infection	Use "yes" or "no", case sensitive
gout	Use "yes" or "no", case sensitive
diabetes	Use "yes" or "no", case sensitive
HTN	Hypertension. Use "yes" or "no", case sensitive
PH	Primary Hyperoxaluria. Use "yes" or "no", case sensitive
Cystinuria	Use "yes" or "no", case sensitive
Smoking	Use "yes" or "no" or "former", case sensitive
Alcohol	Use "yes" or "no", case sensitive
Frequent_urination	Use "yes" or "no", case sensitive
Urgent_urination	Use "yes" or "no", case sensitive

Painful_urination	Use "yes" or "no", case sensitive
Hesitancy	Use "yes" or "no", case sensitive
Dribbling	Use "yes" or "no", case sensitive
Intermittent_urine_flow	Use "yes" or "no", case sensitive
Back_flank_abdominal_pain	Use "yes" or "no", case sensitive
Urine_blood	Use "yes" or "no", case sensitive
**GI_Variables**	
probiotics	Use "yes" or "no", case sensitive
Probiotic_name	Free response - "Please name the probiotic (s) taken
bowel_movements	"None", "1_2", "3_4", "5_6", "7_8", "8_9", "10_14", "14_" Question - How many bowel movements do you normally have per week?
Constipation_history	Personal history of constipation. Use "yes" or "no" , case sensitive
Inflammatory_Bowel_Disease	Use "yes" or "no", case sensitive
Irritable_Bowel_Syndrome	Use "yes" or "no", case sensitive
Proton_pump_inhibitor_use	Use "yes" or "no" or "former", case sensitive
Colonoscopy	Use "yes" or "no, case sensitive. Question - Have you had a colonoscopy within 2 weeks of providing a sample?
Gastric_bypass_surgery	Use "yes" or "no", case sensitive. Include free text response option for type of surgery and year
GI_Surgery	Use "yes" or "no", case sensitive. Include free text response option for type of surgery and year
**Lab_Variables**	
Latest_lab_value_Ca_mg_dL	Calcium. Numerical value
Latest_lab_value_Ph_mg_dL	Phosphate. Numerical value
Latest_lab_value_PTH_ng_L	Parathyroid hormone. Numerical value
Latest_lab_value_Uric_acid_mg_dL	Numerical value
Latest_lab_value_Ox_mg_dL	Oxalate. Numerical value
Latest_lab_value_pH	pH. Numerical value
Latest_lab_value_Cr_mg_dL	Creatinine. Numerical value
24hr_urine_chemistry	Times Performed "0", "1", "2", "3_"
**Diet_Variables**	
diet_type	Use "Low_Carbohydrate", "Omnivore", "Pescaterian", "lacto-vegetarian," "ovo-vegetarian," lacto-ovo-vegetarian," "pesca-lacto-vegetarian," "pesca-ovo-vegetarian," "pesca-lacto-ovo-vegetarian," "vegetarian_no_dairy_ no_eggs)", "Vegan", "Keto", "DASH" Other diets can easily be added. Case sensitive
meals per day	"None", "1"_"2"_"3"_"4"_"5 or more". Question – How many meals do you usually eat in a day?

snacks	Use "yes" or "no, case sensitive. Question – Do you normally snack between meals on most days?
dairy_NOT_including_cheese_or_cottage_cheese	"None", "1"_"2"_"3"_"4"_"5 or more". Question - How many times a day to you eat or drink yogurt, pudding made with milk, kefir, cow's milk, goat's milk or some other animal milk?
cheeses	"None", "1"_"2"_"3"_"4"_"5 or more". Question - How many times a day to you eat cheese (can also ask for ounces/day as some people know this) or cottage cheese (for cottage cheese, can ask cups/day)?
nuts_seeds_and_nut_seed_butters	"None", "1"_"2"_"3"_"4"_"5 or more". Question - How many times a day to you eat nuts or seeds or use a nut butter or seed butter? (Some people may be able to quantify in cups/day)
packaged_foods__prepared_foods	"Mostly_packaged_or_prepared_foods," "occasionally_packaged_or_prepared_foods," or "rarely_or_never_packaged_or_prepared_foods" for the question - "Would you describe your usual diet as comprised of mostly packaged or prepared foods, occasional packaged or prepared foods, or little to no packaged or prepared foods?
water	Numerical value for amount in ounces or liters/day
total_fluids_includes_water_and_also_other_beverages	Numerical value for estimated ounces or liters/day
dessert	"yes" or "no" for the question - "Do you eat sweets, candies, pastries, or desserts daily?"
sugary_beverages_and_cereals	"yes" or "no" for the question - "Do you drink sugar-sweetened beverages (such as soda, fruit drinks, fruit punch, kool-aid, sweet tea, sweetened coffee drinks) or sugar-sweetened cereals daily?"
meat	"None", "1_2", "3_4", "5_6", "7_8", "8_9", "10_14", "14_". Question - How many times per week do you eat meat?
processed_meats	"None", "1_2", "3_4", "5_6", "7_8", "8_9", "10_14", "14_". Question - How many times per week to you eat processed meats, such as cold cuts, sliced lunch meats, bacon, sausage, jerky, ham?
fruit	"None", "1_2", "3_4", "5_6", "7_8", "8_9", "10_". Question - How many servings of fruit do you eat each day? If you can't define a "serving," then how many times a day do you typically eat fruit?
veggie	"None", "1_2", "3_4", "5_6", "7_8", "8_9", "10_". Question - How many servings of vegetables do you eat each day? If you can't define a "serving," then how many times a day do you typically eat a vegetable?
bread	"None", "1_2", "3_4", "5_6", "7_8", "8_9", "10_". Question - How many slices of bread or muffins or bagels or rolls do you usually have in a day?
eating_out__carrying-out	"None", "1_2", "3_4", "5_6", "7_8", "8_9", "10_". Question - How many times in a week do you typically eat out or carry in a meal from a restaurant?

Urine Sample Collection Instructions

Dear Participant,

Thank you for participating in our study and for your agreement to collect a urine sample from you.

Please collect and mail the urine sample as per the instructions on the following pages for collection and shipment of the urine sample. Please aim to provide the urine sample within 72 hours (3 days) of the stool sample you are also providing, if possible.

Please do not remove the Styrofoam insert from the cardboard shipping box. If you are not shipping your sample immediately, place the sealed biohazard bag with the urine tubes in your refrigerator until shipment. The urine samples cannot be frozen.

If you have any questions or concerns during this process, please contact the Study Coordinator.

Thank you again for your very valuable contribution to our study!

Materials for Collection/Shipment of Urine Sample

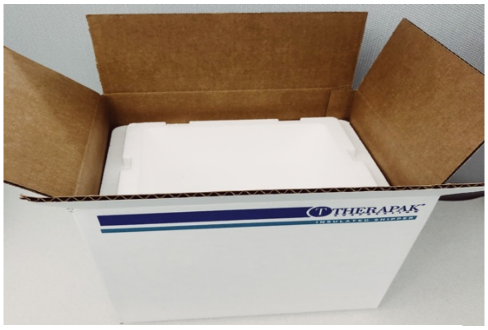

Styrofoam cooler (to be kept inside cardboard box)

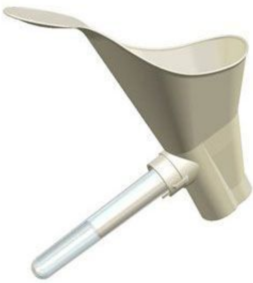

Peezy urine collection with boric acid tubes

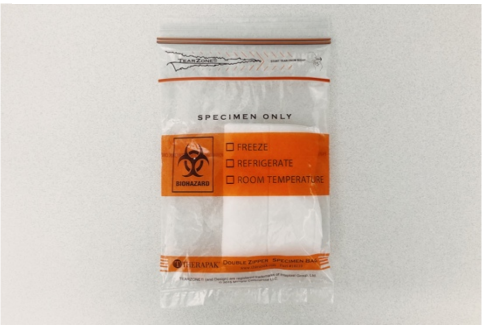

Biohazard bag with absorbent

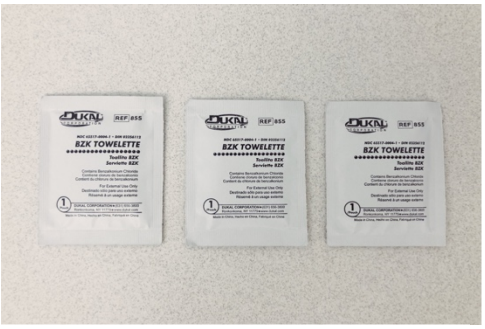

Antiseptic wipes

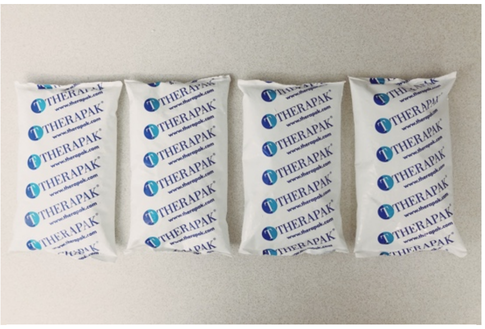

Ice packs

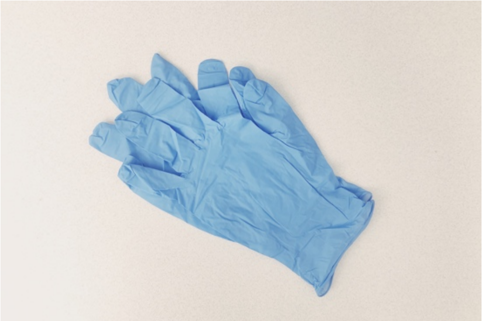

Gloves

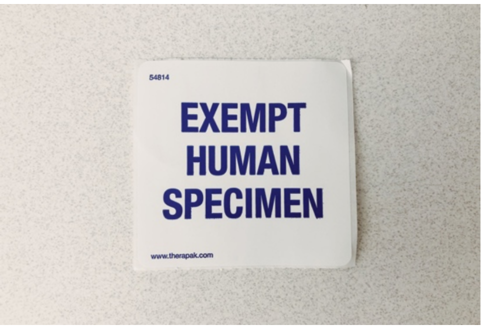

Exempt Human Specimen sticker

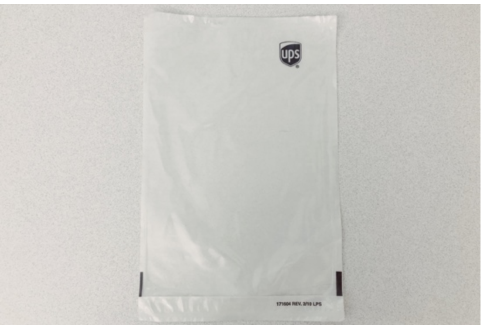

Plastic address pouch with return label

Instructions for Collection of Urine Sample

1. Immediately after receiving the urine collection kit, place the ice packs flat in your freezer.
2. You have been provided with a pair of sterile gloves and a sterile peezy urine collection system. Please put the gloves on before collecting your urine specimen.
3. Wipe the genital region with the wipe provided.
   A. Males: Please cleanse the tip of the penis with the antiseptic wipe beginning at the urethral opening and then working away from the end and towards your body. If you have foreskin, please temporarily pull back the foreskin.
   B. Females: Please cleanse the area around the vaginal opening with the antiseptic wipe.
4. Collect the urine sample:
   Remove peezy collection system from packaging and follow the instructions on packaging to collect urine. Video instructions are available at https://forte-medical.co.uk/midstream/. Try to fill the bottle to the 20ml line. After removing the bottle for the collection system, replace the cap onto the bottle. The peezy collection system can be disposed in the waste.
5. **Fill out the information on the label and make sure to attach it to each tube. Fill out the “Date Collected” and “Time collected” lines on the Urine Sample Collection Form**.
6. Place bottle into the provided biohazard bag. Seal biohazard bag.
7. Put the biohazard bag into the box with the ice packs provided.
8. When you are ready to ship the urine sample, please refer to instructions on following page.

Instructions for Shipping of Urine Sample

Please read the following important points before shipping the urine sample:

- The urine sample should be shipped as soon as possible after collection and must reach us within 24 hours of collection.
- The best days to ship the urine sample are Mondays, Tuesdays, and Wednesdays. Samples cannot be received on weekends or holidays.

1. Contact the Study Coordinator to schedule the USPS pickup from your house.

2. Place the sealed biohazard bag with the urine sample in the Styrofoam cooler.

3. Place the four frozen ice packs around the biohazard bag with the urine sample so that it is completely surrounded.

4. Place the lid on the Styrofoam cooler.

5. Place the completed Urine Sample Collection Form on top of the closed Styrofoam cooler.

6. Seal the cardboard shipping box with packing tape.

7. Place the “Exempt Human Specimen” sticker on the outside of the cardboard shipping box.

8. Place the return label on the outside of the cardboard shipping box. The return label is located in the plastic address pouch.

9. Place the cardboard shipping box outside of your home for pickup by USPS. The Study Coordinator will schedule the pickup.

Urine Sample Collection Instructions

Instructions for Collection of Urine Sample – catheterized or upper tract urine

These instructions are for urine specimens collected by:

A. Bladder catheterization
B. Upper urinary tract urine (at time of ureteroscopy or percutaneous nephrolithotomy) via direct aspiration of renal pelvis urine with a syringe

1. Maintain sterility using a pair of sterile gloves and the sterile urine collection cup provided.

2. Collect the urine sample:
   Collect the urine in the sterile cup (if via sterile catheterization approach of bladder) or transfer the collected urine in the syringe (upper urinary tract urine) into the collection cup. Do not touch the inside of the cup. Collect as much urine as possible.

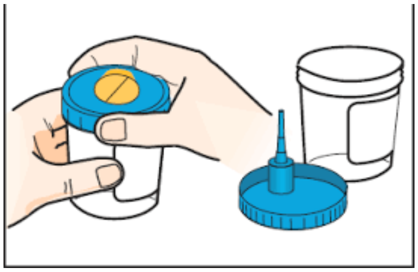

3. After collecting the urine sample, place the cup upright on clean, flat surface.

4. **As soon as possible**, peel back the label on the cap of the collection cup (blue cap) to expose the integrated transfer device. The transfer device looks like a needle.

5. Take the tube with the **GREY** cap, which contains boric acid as a preservative. Turn it upside down and with the stopper down, place it on top of the needle transfer device on the blue cap collection cup.

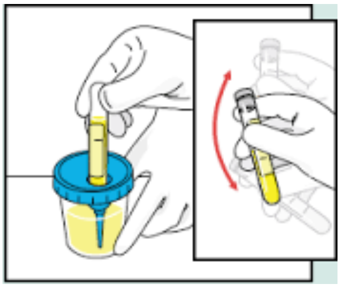

6. Advance the grey cap tube over puncture point to pierce stopper.
7. Hold the tube in position until filled.
8. Remove grey top tube from integrated transfer device.
9. Mix the tube 8 - 10 times by inversion i.e. turning it upside down.
10. Next repeat these steps with the tube with the **YELLOW** cap.
11. Take the tube with the yellow cap, turn it upside down and with the stopper facing down, place the stopper on the needle transfer device of the blue cap collection cup. The collection cup should still be kept flat on a hard surface.
12. Advance the yellow top tub over the puncture point to pierce the stopper.
13. Hold the tube in this position until it is filled.
14. Replace the label over the integrated transfer device cavity and reseal. Use caution to avoid contact with needle when replacing label.
15. **Fill out the information on the label and make sure to attach it to each tube. Fill out the "Date Collected" and "Time collected" lines on the Urine Sample Collection Form**.
16. Place grey and yellow tubes into the provided biohazard bag. Seal biohazard bag.
17. Put the biohazard bag into the box with the ice packs provided.
18. Transfer specimens to research lab as soon as possible after completion of the procedure. If unable to transfer immediately, samples can be preserved in AssayAssure and placed the sealed biohazard bag in the refrigerator until taken to lab (must be within 24 hours of collection).

Urine sample collection form

Please DO NOT record names of study participant anywhere on this form.

Participant ID: ___________________

Urine Sample:

Date Sample Collected: _____________

Time Sample Collected: _____________ AM / PM *(please circle)*

For Lab Use Only:

Date Delivered: _____________

Time Delivered: _____________ AM / PM (please circle)

Was sample temperature maintained during shipment? ☐ Yes ☐ No

Comments about sample condition at arrival:

Sample type: _____________

Sample timepoint: _____________

Stool Sample Collection Instructions

Dear Participant,

Thank you for participating in our study and for your agreement to collect a stool sample from you.

Please collect and mail one stool sample as per the instructions on the following pages for collection and shipment of the stool sample. Please aim to provide the stool sample within 72 hours (3 days) of the urine sample you are also providing, if possible.

Please do not remove the Styrofoam cooler from the cardboard shipping box. If you are not shipping your sample immediately, place the sealed Ziploc bag with the stool sample upright in your refrigerator until shipment. The stool sample cannot be frozen.

If you have any questions or concerns during this process, please contact the Study Coordinator.

Thank you again for your very valuable contribution to our study!

<u>Materials for Collection/Shipment of Stool Sample</u>

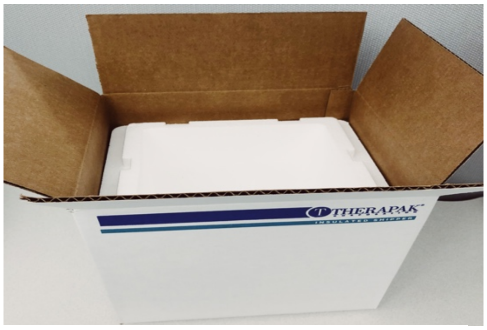

Styrofoam cooler (to be kept inside cardboard box)

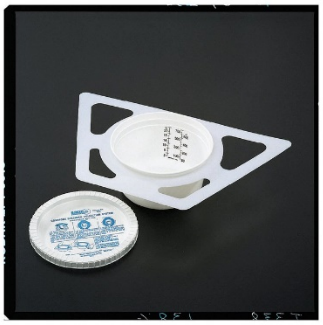

Stool collection bucket with lid

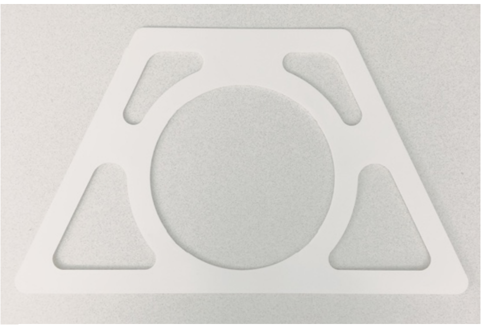

Bucket frame

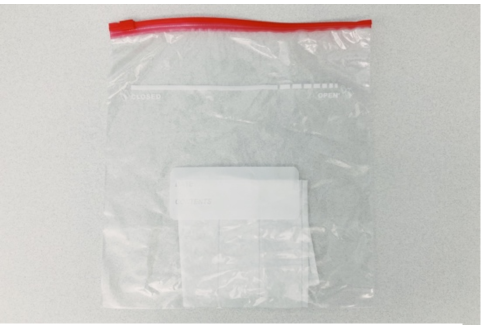

Ziploc bag with absorbent

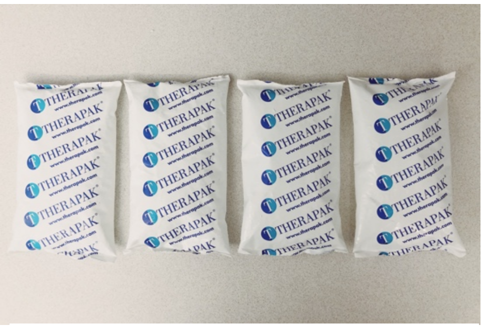

Ice packs

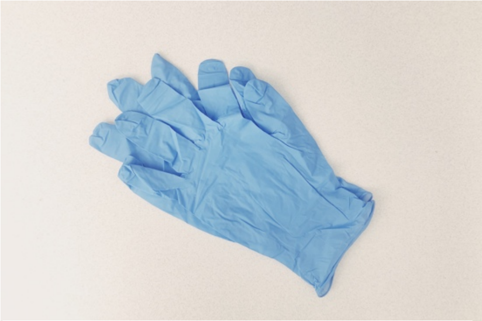

Gloves

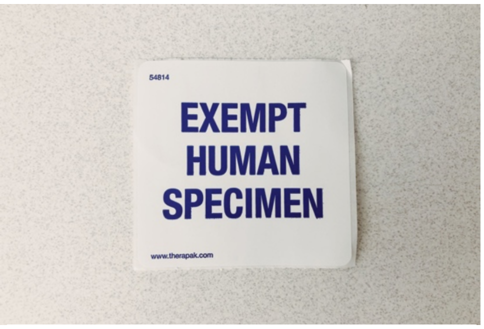

Exempt Human Specimen sticker

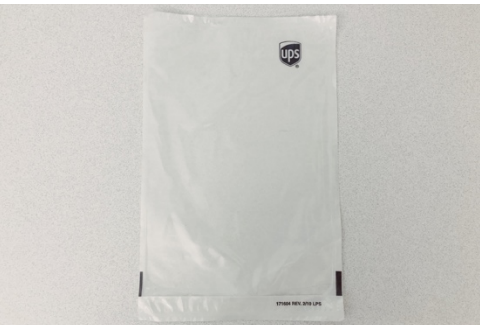

Plastic address pouch with return label

<u>Instructions for Collection of Stool Sample</u>

1. Immediately after receiving the stool collection kit, place the ice packs flat in your freezer for at least 12 hours.
2. You have been provided with a pair of sterile gloves and a sterile fecal collection kit.
3. Preparing for stool sample collection:
   A. Before having a bowel movement, put the gloves on.
   B. Retrieve the bucket frame.
   C. Lift up the toilet seat and place the bucket frame under the toilet seat.
   D. Set the toilet seat back down and place the stool collection bucket inside the frame.

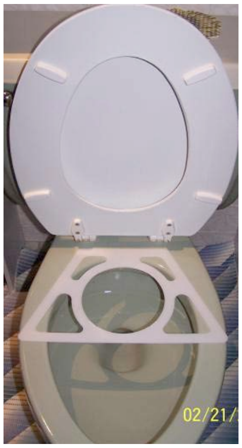
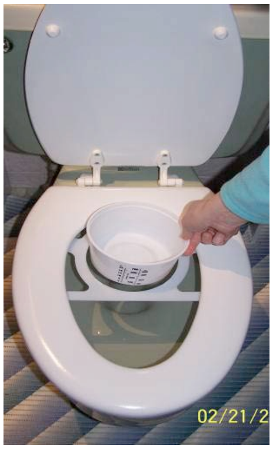
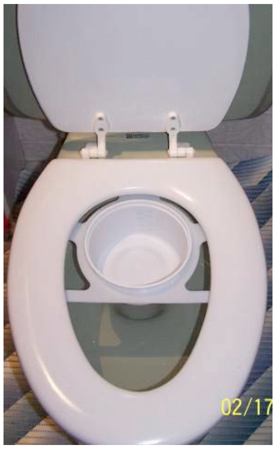

4. Collecting the stool sample:
   A. Have bowel movement as normal into collection bucket, ensuring that no urine or waste tissue gets into the container. Try to collect as much stool as possible.
   B. After the bowel movement, put the gloves on and remove the container containing the stool and place the lid onto the container.
5. Place closed container into the provided Ziploc bag and seal the Ziploc bag.
6. Discard the collection frame in the trash.
7. **Fill out the Stool Sample Collection Form.**
8. Put sample back into the box with the ice pack provided.
9. When you are ready to ship the stool sample, please refer to the instructions on the following page

Instructions for Shipment of Stool Sample

Please read the following important points before shipping the stool sample:

- The stool sample should be shipped within 24 hours of collection.
- The best days to ship the stool sample are Mondays, Tuesdays, and Wednesdays. Samples cannot be received on weekends or holidays.

1. Contact the Study Coordinator to schedule the UPS pickup from your house.

2. Place the sealed Ziploc bag with the stool sample in the Styrofoam cooler.

3. Place the four frozen ice packs around the Ziploc bag containing the stool sample so that it is completely surrounded.

4. Place the lid on the Styrofoam cooler.

5. Place the completed Stool Sample Collection Form and Baseline Information Case Report Form on top of the closed Styrofoam cooler.

6. Seal the cardboard shipping box with packing tape.

7. Place the “Exempt Human Specimen” sticker on the outside of the cardboard shipping box.

8. Place the return label on the outside of the cardboard shipping box. The return label is located in the plastic address pouch.

9. Place the cardboard shipping box outside of your home for pickup by UPS. The Study Coordinator will schedule the pickup.

Stool sample collection form

Please DO NOT record names of study participant anywhere on this form.

Participant ID: ____________________

Stool Sample:

Date (*mm/dd/yyyy*) Sample Collected: ______________

Time Sample Collected: ______________ AM / PM *(please circle)*

Where was the stool collected? ☐ Home ☐ Hospital ☐ Other: _________

Did the subject have a previous bowel movement on day of collection?:

☐ Yes ☐ No ☐ Unknown

For Lab Use Only:

Date Delivered: ______________

Time Delivered: ______________ AM / PM *(please circle)*

Was sample temperature maintained during shipment? ☐ Yes ☐ No

Comments about sample condition at arrival:

Sample timepoint: ______________

Stone Sample Collection Protocol

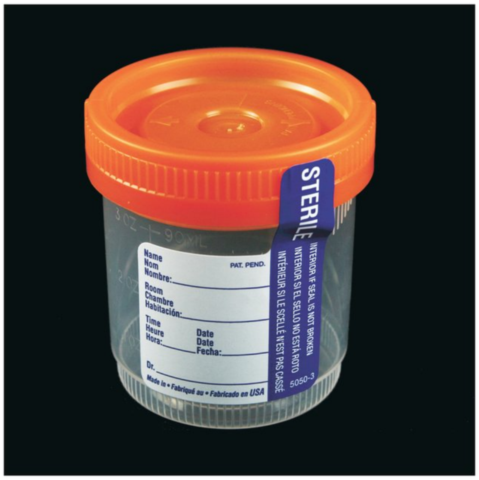

Sterile specimen collection cup

1. For stone samples collected during surgical procedures (ureteroscopy or percutaneous nephrolithotomy), transfer a minimum of 500mg aseptically to a sterile specimen cup. The remaining stone samples can be sent for clinical analysis of composition or other analyses. For ureteroscopy, utilization of ureteral access sheath is preferred to avoid bladder/urethral contamination during passage of ureteroscope and calculus.
2. Stone samples should be transferred to 2ml Eppendorf tubes and frozen at -80ºC within two hours, prior to DNA extraction or be used directly for DNA extraction.
3. Stone samples should be rinsed thoroughly with sterile PBS to remove surface bacteria.
4. After rinsing, flash freeze stone sample in liquid nitrogen and pulverize to a fine powder with a bullet blender. Thoroughly rinse the blender with bleach between samples. Sterile PBS should also be processed in the bullet blender and put through the remaining workflow as a negative control.
5. DNA extraction should proceed with an automated DNA extraction machine such as a KingFisher Duo Prime (Fisher), QiaCube (Qiagen), or similar machine using

protocols designed for urine or soil samples. DNA extraction protocols should include enzymatic lysis, such as with Proteinase K, lysozyme, or Mutanolysin.

6. Negative controls should include sterile PBS and positive controls should include a mixed microbial community DNA sample that is run with every sequencing batch. If this is your first time with high-throughput sequencing, combine a small aliquot ~2-3μl from the DNA of each of your samples to be used as a positive control for this and future batches of sequencing. Controls help to minimize technical biases between batches.
7. After extraction, DNA should be stored at -80ºC.
8. Sequencing should proceed at a sequencing facility using either primers for the V4 region of the 16S rRNA gene on an Illumina MiSeq machine (16S sequencing) or on a HiSeq, NextSeq, or NovaSeq (Shotgun metagenomics).

Urine processing for investigators

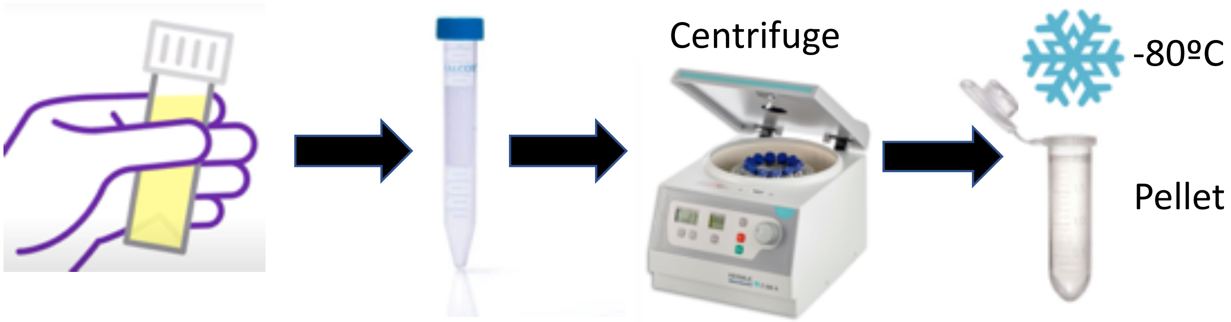

IMPORTANT NOTES/CONSIDERATIONS

- Human urine samples should be considered a BSL 2 material. Samples must be handled in a BSL 2 approved facility and disposed of in accordance to BSL 2 standards.
- Samples should be maintained at cool temperature (4ºC) and avoid freeze-thaw cycles prior to collection.
- Samples should be stored at -80ºC after collection in a BSL 2 approved

I) Required PPE

- Biological Safety Cabinet (BSC)
- Safety googles
- Disposable lab coat (or lab coat dedicated to collection)
- Disposable sleeve covers
- Gloves
- Face mask

II) Required Collection Supplies

- Urine sample
- Self-Sealing Sterilization Pouches (Fisher 01-812-51)
- 15mL conical tubes (Fisher 14-959-70C)
- 1.5mL microcentrifuge tubes (USA Scientific 1615-5500)
- AssayAssure®
- Bleach
- Spill paper
- Freezer boxes
- Paper towels
- Sharpie

III) Collection Procedure Preparation

1. Spray down BSC with prepared Bleach spray and wipe clean.

2. Lay down spill paper and mist with Bleach spray.

3. Bring supplies into the BSC.
   - Sharpie
   - 15mL conical tubes

4. Label 15mL conical tubes and 1.5mL microcentrifuge tube with subject ID.

5. Transfer the urine sample from the collection tube to the 15mL conical tube. An aliquot of urine can be frozen at -80ºC for metabolomic or other assays.

6. Centrifuge urine sample at 5000rpm for 5 minutes.

7. Carefully decant the supernatant without disturbing the pellet at the bottom of the tube.

8. Transfer ~1ml of the pellet to labeled 2ml Eppendorf tube and store at -80ºC until DNA extraction or can be used for DNA extraction directly.

9. Centrifuge urine samples at 14000rpm for 5 minutes.

10. With a pipettor, collect the pellet at the 400μl setting.

11. DNA extraction should proceed with an automated DNA extraction machine such as a KingFisher Duo Prime (Fisher), QiaCube (Qiagen), or similar machine using protocols designed for urine or soil samples.

12. Negative controls should include sterile PBS and positive controls should include a mixed microbial community DNA sample that is run with every sequencing batch. If this is your first time with high-throughput sequencing, combine a small aliquot ~2-3μl from the DNA of each of your samples to be used as a positive control for this and future batches of sequencing. Controls help to minimize technical biases between batches.

13. After extraction, DNA should be stored at -80ºC.

14. Sequencing should proceed at a sequencing facility using either primers for the V4 region of the 16S rRNA gene on an Illumina MiSeq machine (16S sequencing) or on a HiSeq, NextSeq, or NovaSeq (Shotgun metagenomics).

Stool processing for investigators

Fecal Collection (via FAST) SOP

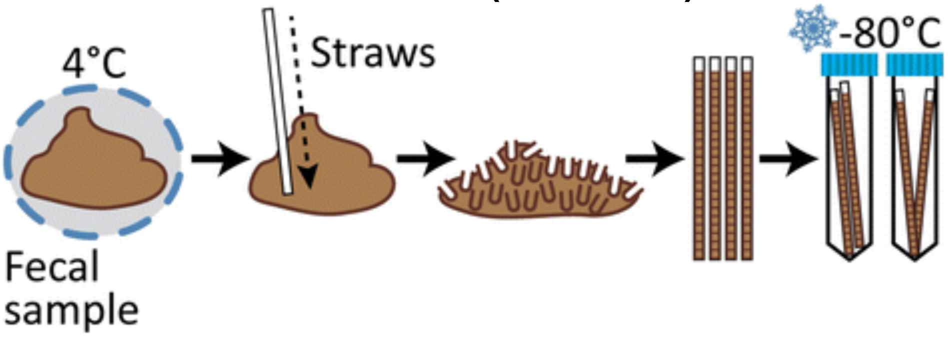

For more detail about collection procedures and sample usage, reproducibility, or viability please see the following article:
https://microbiomejournal.biomedcentral.com/articles/10.1186/s40168-018-0458-8

IMPORTANT NOTES/CONSIDERATIONS

- Human fecal samples are a BSL 2 material. Samples must be handled in a BSL 2 approved facility and disposed of in accordance to BSL 2 standards.
- Samples should be maintained at cool temperature (4ºC) and avoid freeze-thaw cycles prior to collection.
- Samples should be stored at -80ºC after collection in a BSL 2 approved freezer.

I) Required PPE

- Biological Safety Cabinet (BSC)
- Safety googles
- Disposable lab coat (or lab coat dedicated to collection)
- Disposable sleeve covers
- Gloves
- Face mask

II) Required Collection Supplies

- Stool sample container (Fischer 02-544-208)
- Smart Spatula (Sigma Z560049-300EA)
- Self-Sealing Sterilization Pouches (Fischer 01-812-51)
- 15mL conical tubes (Fischer 14-959-70C)
- 1.5mL microcentrifuge tubes (USA Scientific 1615-5500)
- Trifectant tablets (Amazon)
- Spill paper
- Freezer boxes
- Paper towels
- Dry ice
- Sharpie

III) Collection Procedure Preparation

Prior to sample collection the following step will need to be taken.

1. Prepare “straws” for autoclaving.
    - Cut off the two ends of the spatula (dashed lines) then cut the spatula in half (solid line). Each half is referred to as a straw from here on.

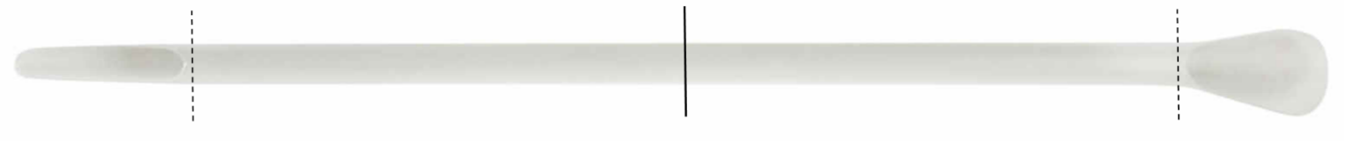

    - Place the desired number of straws per subject (recommended minimum is 4) and a single 15mL tube into a serializable pouches and autoclave at 121ºC for 30 minutes of sterilization plus 20 minutes of drying time on the dry setting.

IV) Collection Procedure Preparation

15. Spray down BSC with prepared Trifectant spray and wipe clean. NOTE: Prepared trifectant spray is good for one week from reconstitution.

16. Lay down spill paper and mist with Trifectant spray.

17. Bring supplies into the BSC.
    - Dry ice
    - Sharpie
    - Autoclaved straws
    - 15mL conical tubes

18. Label 15mL conical tubes and 1.5mL microcentrifuge tube with subject ID. Place on dry ice.

19. Open sample container. Repetitively and vigorously insert the straw into the sample in a random and dispersed manned. This action should <u>NOT</u> result in a “coring” of the sample but rather the backup of a small amount of sample each time. Leave a fingers width opening at the top of the straw, DO NOT OVERFILL.

20. Clean off any material on the outside of the straw into the 1.5mL microcentrifuge tube. This can be used for DNA extraction for 16S analysis purposes.

21. Place the cleaned straw into the 15mL conical and store on dry ice.

22. Repeat **Step 5** with the remaining straws. NOTE: 2 straws will fit into a single 15mL conical tube.

23. Between samples:
    - Remove/replace any visibly soiled material (e.g. spill paper or sleeve covers)
    - Change gloves
    - Bring in new straw packet

- Mist spill paper with Trifectant spray

24. Once all samples have been processed transfer from dry ice to the -80ºC freezer or can proceed to DNA extraction. Dispose of remaining samples in accordance with approved protocols.

25. For DNA extraction, using an bleach sterilized metal spatula, collect ~50mg of material from each of the 4 straws (200mg total).

26. DNA extraction should proceed with an automated DNA extraction machine such as a KingFisher Duo Prime (Fisher), QiaCube (Qiagen), or similar machine using protocols designed for stool samples.

27. Negative controls should include sterile PBS and positive controls should include a mixed microbial community DNA sample that is run with every sequencing batch. If this is your first time with high-throughput sequencing, combine a small aliquot ~2-3μl from the DNA of each of your samples to be used as a positive control for this and future batches of sequencing. Controls help to minimize technical biases between batches.

28. After extraction, DNA should be stored at -80ºC.

29. Sequencing should proceed at a sequencing facility using either primers for the V4 region of the 16S rRNA gene on an Illumina MiSeq machine (16S sequencing) or on a HiSeq machine (Shotgun metagenomics).
